# Genetic ablation of ketohexokinase C isoform impairs pancreatic cancer development

**DOI:** 10.1016/j.isci.2023.107368

**Published:** 2023-07-13

**Authors:** Ilaria Guccini, Guanghui Tang, Trang Thuy To, Laura Di Rito, Solange Le Blanc, Oliver Strobel, Mariantonietta D’Ambrosio, Emiliano Pasquini, Marco Bolis, Pamuditha Silva, Hasan Ali Kabakci, Svenja Godbersen, Andrea Alimonti, Gerald Schwank, Markus Stoffel

**Affiliations:** 1 Institute of Molecular Health Sciences, ETH Zurich, 8093 Zurich, Switzerland; 2 Computational Oncology Unit, Department of Oncology, Istituto di Richerche Farmacologiche 'Mario Negri' IRCCS, 20156 Milano, Italy; 3 European Pancreas Center, Department of General Surgery, Heidelberg University Hospital, Heidelberg, Germany; 4 Institute of Oncology Research (IOR), Oncology Institute of Southern Switzerland (IOSI), 6500 Bellinzona, Switzerland; 5 Universita’ della Svizzera Italiana, 6900 Lugano, Switzerland; 6 Bioinformatics Core Unit, Swiss Institute of Bioinformatics, TI, 6500 Bellinzona, Switzerland; 7 Department of Medicine, University of Padua, 35128 Padua, Italy; 8 Department of Health Sciences and Technology (D-HEST) ETH Zurich, 8093 Zurich, Switzerland; 9 Institute of Pharmacology and Toxicology, University of Zurich, 8057 Zurich, Switzerland

**Keywords:** Natural sciences, Biological sciences, Biochemistry, Systems biology, Cancer systems biology

## Abstract

Although dietary fructose is associated with an elevated risk for pancreatic cancer, the underlying mechanisms remain elusive. Here, we report that ketohexokinase (KHK), the rate-limiting enzyme of fructose metabolism, is a driver of PDAC development. We demonstrate that fructose triggers KHK and induces fructolytic gene expression in mouse and human PDAC. Genetic inactivation of *KhkC* enhances the survival of *KPC*-driven PDAC even in the absence of high fructose diet. Furthermore, it decreases the viability, migratory capability, and growth of *KPC* cells in a cell autonomous manner. Mechanistically, we demonstrate that genetic ablation of KHKC strongly impairs the activation of KRAS-MAPK pathway and of rpS6, a downstream target of mTORC signaling. Moreover, overexpression of KHKC in *KPC* cells enhances the downstream KRAS pathway and cell viability. Our data provide new insights into the role of KHK in PDAC progression and imply that inhibiting KHK could have profound implications for pancreatic cancer therapy.

## Highlights

• Fructose enhances proliferation and decreases the survival of mice with PDAC
• KHKC overexpression promotes pancreatic tumor growth
• Pancreatic ablation of *KhkC* dampens the proliferation of *KPC* driven tumors
• Pancreatic *KhkC* inactivation rewires PDAC metabolism-related pathways

## Graphical abstract

**Figure:**
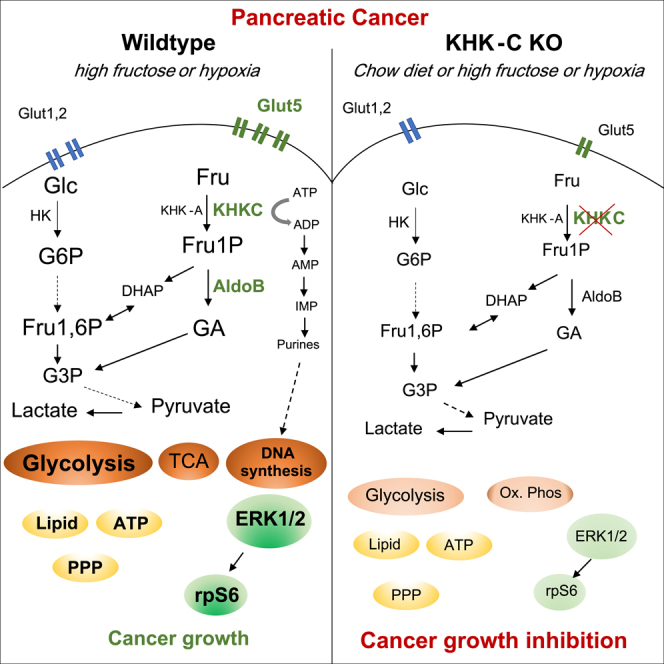

## Introduction

Pancreatic ductal adenocarcinoma (PDAC) is an aggressive tumor and has one of the poorest outcomes, as it is often diagnosed at an advanced stage due to the lack of symptoms. It is projected to be the second leading cause of cancer death by 2030, with the lowest 5-year survival rate of all cancers.^1^^,^^2^ At the genetic level, around 80% of patients carry an oncogenic *KRAS* mutation at an early stage of tumor development, followed by loss of function mutations in the tumor suppressors *TP53*, *SMAD4*, and *CDKN2A* (between 90% and 50%) leading to PDAC.^3^

PDAC tumors display a marked metabolic phenotype and acquire metabolic plasticity to improve cellular fitness to provide a selective advantage for cancer cells during tumorigenesis.^4^^,^^5^ Constitutive activation of KRAS plays a key role in metabolic reprogramming, in particular, the glycolytic switch to support the synthesis of tumor biomass.^6^ Thus, understanding how metabolism is reprogrammed in pancreatic cancer may provide a strategy for innovative interventional therapies. Carbohydrates serve as energy source for production of building blocks through enhanced glycolysis that sustain uncontrolled proliferation of cancer cells. The intake of dietary sugar has increased dramatically in the Western world during the past four decades and has been paralleled by an increased prevalence for diabetes, obesity, cardiovascular disease, and cancer, implying a possible causal relationship.^7^^,^^8^^,^^9^^,^^10^^,^^11^ However, the contribution of specific carbohydrates to disease states is not fully understood. Fructose and glucose are major components of dietary carbohydrates such as sucrose (containing 50% glucose and 50% fructose) and high fructose corn syrup (HFCS, containing 55% fructose and 42% glucose). Glucose can be used directly by various tissues as an energy source or, when in excess, stored in the liver as glycogen or converted into fructose by the polyol pathway.^12^ Low doses of fructose are primarily cleared by the liver (90%),^10^^,^^13^ while high doses of fructose (≥1 g/kg) overwhelm intestinal fructose absorption and clearance, resulting in fructose reaching both the liver and colon, where it can affect gut microbiota and lead to the deterioration of intestinal barrier function.^14^^,^^15^^,^^16^ This in turn can cause endotoxemia, inflammation in the liver, and induction of *de novo* lipogenesis.^17^ The main difference between glucose and fructose metabolism is that glycolysis is tightly regulated according to the cellular energy state at the level of phosphofructokinase (PFK), while fructose degradation to triosephosphates is unrestricted.^18^ The lack of inhibitory feedback mechanisms promotes efficient conversion of fructose into fat. In addition, by limiting ATP levels and restricting ATP-mediated negative feedback inhibition of PFK, unrestrained KHK-driven phosphorylation of fructose can also facilitate high glycolytic flux.^19^ Ketohexokinase (KHK) is the rate-limiting first enzyme of fructose metabolism that converts fructose to fructose-1-phosphate (F1P), which is then further metabolized by aldolase B to enter glycolysis.^20^ KHK exists as a high affinity KHK-C and a low affinity KHK-A isoform that are generated by mutually exclusive splicing of exons 3C and 3A, respectively, of the *KHK* gene.^21^ Alternative splicing of KhkC in the liver is mediated by APOBEC1 complementation factor (A1CF), while heterogeneous nuclear ribonucleoprotein H1/2 (hnRNPH1/2) mediates a c-myc-driven switch of KHKC to the KHKA isoform in dedifferentiated hepatocellular carcinoma (HCC) cells.^16^^,^^22^

Recent studies have shown that fructose consumption is linked to many different types of cancers. Increased fructose uptake, through GLUT5 upregulation, contributes to reinforcing glycolysis in human colorectal, breast, lung cancer, and glioma as well as pancreatic cancer, triggering cell survival and proliferation.^12^^,^^23^ In intestinal cancer, a daily small dose of high-fructose corn syrup enhanced tumor growth in adenomatous polyposis coli (APC)-mutant mice, independent of obesity or metabolic syndrome.^24^ Similarly in acute myeloid leukemia and in prostate cancer, fructose uptake increased cancer cell proliferation and migration *in vitro* and tumor growth *in vivo*.^25^^,^^26^^,^^27^ Loss of KHK function is associated with neoplastic disease, as lower KHK transcript levels or reduced KHK enzymatic activity has been reported in most cancers. However, studies investigating the cell autonomous impact of different KHK-isoforms on cancer cells in connection with fructose metabolism are lacking.

In this study, we dissected the role of different KHK isoforms and fructose metabolism in the development and progression of pancreatic cancer. We demonstrate that KHK is upregulated in pancreatic cancer and show that genetic ablation of *KHKC* is sufficient to delay the onset and the development of PDAC *KPC*-driven PDAC *in vivo* by downregulating MAPK and mTOR signaling. Genetic ablation of the *KHKA* isoform in turn triggers a reprogrammed transcriptional and metabolic profile that confers higher proliferation than *KPC;KhkC*^*−/−*^, *KPC;KhkA/C*^*−/−*^ as well as *KPC* PDAC tumors. Importantly, we show that blocking KHK efficiently prevents the proliferation and the growth of PDAC tumors independently of a high fructose diet.

## Results

### Ketohexokinase is upregulated in murine and human PDAC

**Figure 1:**
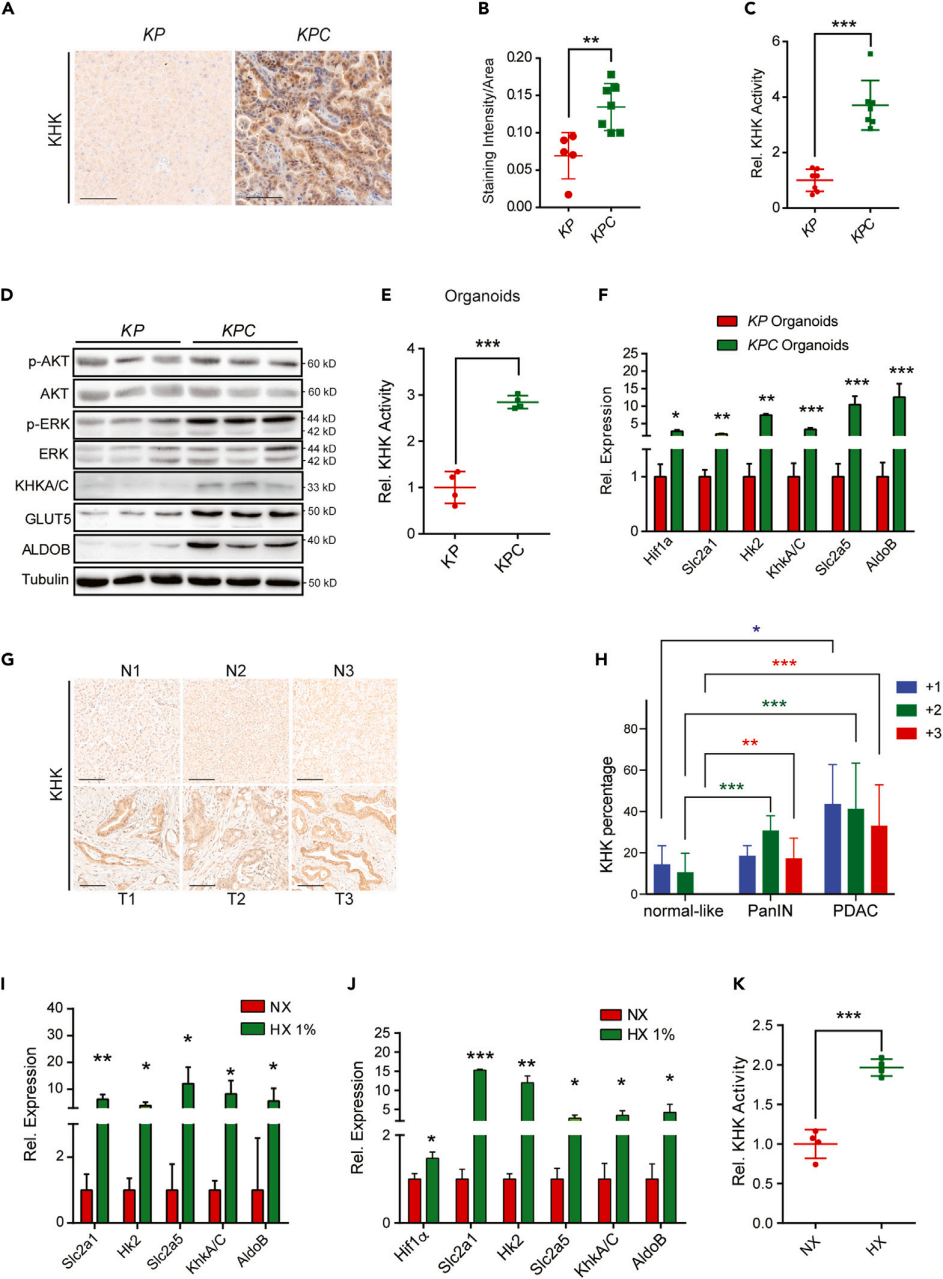
KHKA/C is overexpressed in human and mouse PDAC tumors (A) Representative images of KHK in *KP* versus *KPC* mouse pancreata at the age of 20 weeks. Scale bar: 100 μm. (B) Quantification, represented as mean of staining intensity/area of KHK from *KP* versus *KPC* mouse pancreata, n = 13. (C) KHK activity measured in mouse pancreata, n = 7. (D) Western blots of indicated proteins from *KP* versus *KPC* mouse tumor organoids. Each lane represents an independent tumor organoid. (E) KHK activity determined in *KP* and *KPC* organoids, n = 4. (F) Expression levels of glycolytic and fructose metabolism genes in *KP* and *KPC* mouse organoids, n = 3. (G) Representative IHC images of KHK in human normal pancreata and PDAC tumors. Scale bar: 100 μm. (H) KHK staining intensities as a percentage per area determined by IHC stainings, n = 42. (I) Transcript levels of glycolytic and fructose metabolism genes in human *KPC* organoids in normoxic and hypoxic (1%) conditions, n = 3. (J) Relative transcript levels of glycolytic and fructose metabolism genes in *KPC* organoids, cultured in normoxic and hypoxic (1%) conditions for 24 h, n = 3. (K) KHK activity of *KPC* organoids cultured in normoxic and hypoxic conditions for 24 h, n = 4. Data are represented as Mean ± SEM. The p values were determined by Student’s *t* test (unpaired two-tailed), n.s. (non-significant), ∗p < 0.05, ∗∗p < 0.01 and ∗∗∗p < 0.001.

To determine the relevance of KHK in the exocrine pancreas, we first analyzed the protein levels and compared its expression to organs with known fructose metabolizing function. KHK was highly expressed in the mouse liver and kidney, consistent with the known high fructokinase activity in these organs, while KHK levels in the pancreas were profoundly lower than in the liver and kidney (Figure S1A). To explore the expression and function of KHK in PDAC development, we took advantage of a conditional mouse PDAC model that is based on the exocrine pancreas-specific activation of *Kras*^*G12D*^ and loss of *Trp53* and instigated by *Ptf1α*-*Cre* recombination (*Ptf1α-Cre*; *LSL-KRAS*^*G12D*^; *Trp53*^*fl/*+^, hereafter termed *KPC*).^28^^,^^29^^,^^30^ Interestingly, we measured higher KHK expression in pancreatic tumor biopsies compared to the normal controls (*LSL-KRAS*^*G12D*^; *Trp53*^*fl/*+^, KP) (Figures 1A and 1B). KHK enzymatic activity was also increased (Figure 1C), which likely can be attributed to the upregulation of the KHKC isoform, while the KHKA isoform was not (Figure S1B). Furthermore, we saw a robust upregulation of GLUT5 and ALDOB, and increased phosphorylation of pERK and pAKT (Figure S1B).

We next generated epithelial tumor organoids of *KP* and *KPC* pancreata. This *in vitro* model fully recapitulated the ductal cell lineage characteristics as shown by high Ck19 and Sox9 that were enriched in the *KP* and *KPC* organoids compared to the total pancreatic tissue. Genes associated with the acinar (Ptf1α, Cpa1, and Amy), and endocrine (Ngn3, Chga, and Ins2) lineages were not expressed in either *KP* or *KPC* organoids (Figure S1C). In addition, *KPC* organoids upregulated genes that are indicative of a PanIN disease state (Muc5ac, Muc6, and Tff1) in comparison to *KP* (Figure S1D).^31^ Consistent with the observations in mouse tumors, epithelial *KPC* tumor organoids exhibited higher GLUT5, KHK, ALDOB protein levels, and higher activity of KHK compared to the normal *KP* organoids. In addition, we measured increased levels of glycolytic (Slc2A1 and Hk2) and fructolytic transcripts (Slc2A5, KhkA/C, and AldoB) (Figures 1D–1F).

To determine the expression of KHK in biopsies of patients with pancreatic carcinoma we performed immunohistochemical analysis (IHC) of tissue sections of human tumor and adjacent non-tumor pancreatic tissues in 42 cases. While the majority of tumor tissues stained positive for KHK, the adjacent non-tumor pancreatic tissues displayed low or no positivity (Figures 1G and 1H). Interestingly, patient-derived organoids (PDO) from pancreatic cancer showed overexpression of key glycolytic and fructolytic transcripts upon induction of hypoxia (1%) (Figure 1I). Similarly, key glycolytic and fructolytic transcripts were upregulated, and KHK enzymatic activity was increased in organoids of *KPC* mice upon exposure to hypoxic conditions (Figures 1J and 1K). Together, these data demonstrate that expression of rate-limiting glucose and fructose metabolism genes, including KHK-C, are induced in hypoxic conditions as well as Kras/p53-driven pancreatic cancers of mice and humans.

### High fructose enhances proliferation and decreases the overall survival of mice with *KPC*-driven PDAC

**Figure 2:**
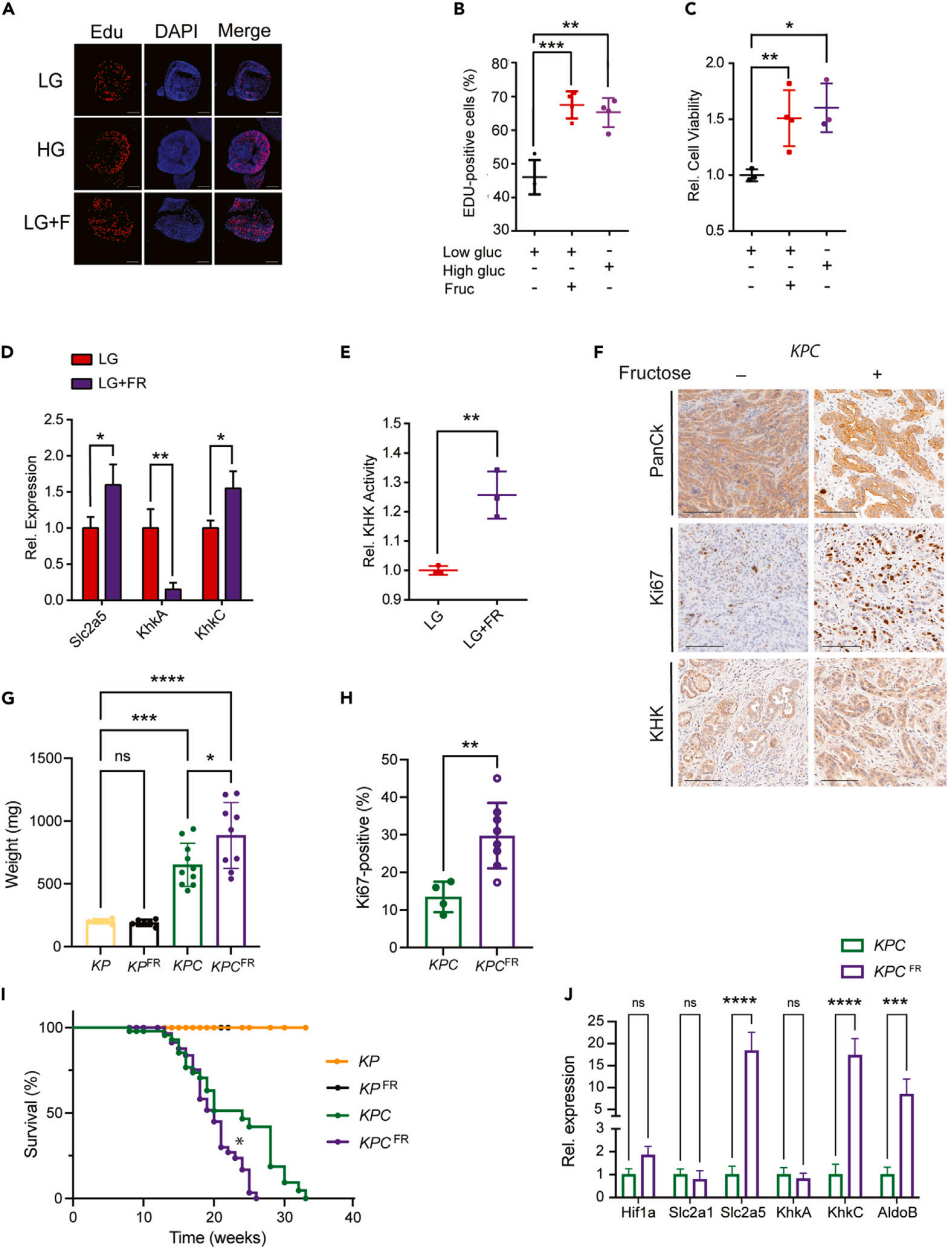
High Fructose enhances proliferation and decreases the overall survival of mice with *KPC*-driven PDAC (A) Representative images of Edu incorporation assay performed in *KPC* organoids and cultured in low glucose (LG, 3 mM), high glucose (HG, 17.3 mM) or low glucose with fructose (LG, 3 mM, 1 mM FR) medium for 48 h. Scale bar: 100 μm. (B) Dot plot representing the percentage of Edu positive cells from organoids as shown in A, n = 3. (C) Dot plot representing the percentage of cell viability from organoids as shown in A, n = 3. (D) Relative transcript levels of Slc2a5, KhkA and KhkC isoforms in *KPC* organoids, cultured in low glucose (LG) and low glucose with 1 mM fructose (LG + FR), n = 3. (E) KHK activity measurements of *KPC* organoids cultured in low glucose (LG) and low glucose with 1 mM fructose (LG + FR), n = 3. (F) Representative IHC images of PanCK (Pan-Cytokeratin), Ki67 and KHK stainings in tissue sections from *KPC* mice treated with 25% of fructose for 10 weeks. Scale bar: 100 μm. (G) Tumors weight endpoints (mg) from *KP* and *KPC* mice treated with 25% of fructose diet for 10 weeks, n = 31 in total. The p values were determined by ANOVA multiple comparison test (Sidák’s multiple comparison test). (H) Percentage of Ki67 positivity in *KPC* mice treated with 25% of fructose diet for 10 weeks, n = 12 in total. The p values were determined by Student’s *t* test (unpaired two-tailed). (I) Survival percentage of *KP* and *KPC* mice with or without 25% of fructose diet for 10 weeks. Kaplan-Meier survival curves were compared by Mantel-Cox log rank test, n = 177 in total. (J) Relative transcript levels of glycolytic (Hif1α, Slc2a1) and fructose metabolic genes (Slc2a5, KhkA, KhkC and AldoB) in *KPC* pancreata treated with a fructose 25% diet, n = 3. Data are represented as Mean ± SEM. The p values were determined by Student’s *t* test (unpaired two-tailed) or ANOVA multiple comparison test (Sidák’s test) when mentioned, n.s. (non-significant), ∗p < 0.05, ∗∗p < 0.01 and ∗∗∗p < 0.001, ∗∗∗∗p < 0.0001.

To investigate the function of KHK and fructose diet in pancreatic cancer development, we first treated mouse-derived organoids with either high (17.3 mM) or low (3 mM) glucose, or low glucose plus high fructose (1 mM). Similar to high glucose, high-fructose treatment enhanced cell proliferation and viability in *KPC* organoids (Figures 2A–2C). Cell viability and growth correlated in a fructose dose-dependent manner in *KPC* compared to control organoids from *KP* mice (Figures S2A and S2B). Interestingly, high fructose treatment (LG + FR) in *KPC* organoids induced the expression of Slc2a5 and KhkC, two rate-limiting proteins for fructose transport into cells and fructose metabolism, while KhkA expression was repressed compared to low glucose (LG) treatment. KHK activity was enhanced in high fructose treatment compared to low glucose (Figures 2D and 2E). Consistent with the *in vitro* data, high-fructose diet in mice (25% in drinking water) enhanced KHK expression, the weight and proliferation rates of PDAC tumors as shown by Ki67-positive cells staining (Figures 2F–2H). Strikingly, a high-fructose diet decreased the overall survival of *KPC,* but not of *KP* mice (Figure 2I). Furthermore, we measured higher plasma fructose levels in both *KP* and *KPC* mice treated with a high fructose diet (Figure S2C). Tumors from mice treated with high fructose revealed higher KhkC expression but similar KhkA levels. Furthermore, Slc2a5 and AldoB transcripts were strongly upregulated upon high dietary fructose exposure (Figure 2J). Similar results were observed in the *KPC* PDAC model harboring the gain-of-function mutation on *Trp53*^*R172H*^ (hereafter termed *KPC*^*mut*^). When exposed to high fructose, these mice, similar to *KPC* animals, exhibited decreased overall survival associated with increased pancreatic tumor burden and higher tumor weight compared to normal diet (Figures S2D–S2I). These results highlight the role of dietary fructose in fructose metabolism, cell proliferation, tumor progression and overall survival in pancreatic cancer *KPC* mice.

### KHKC overexpression promotes tumor growth and *KhkC* KO is sufficient to delay the onset and development of tumors

**Figure 3:**
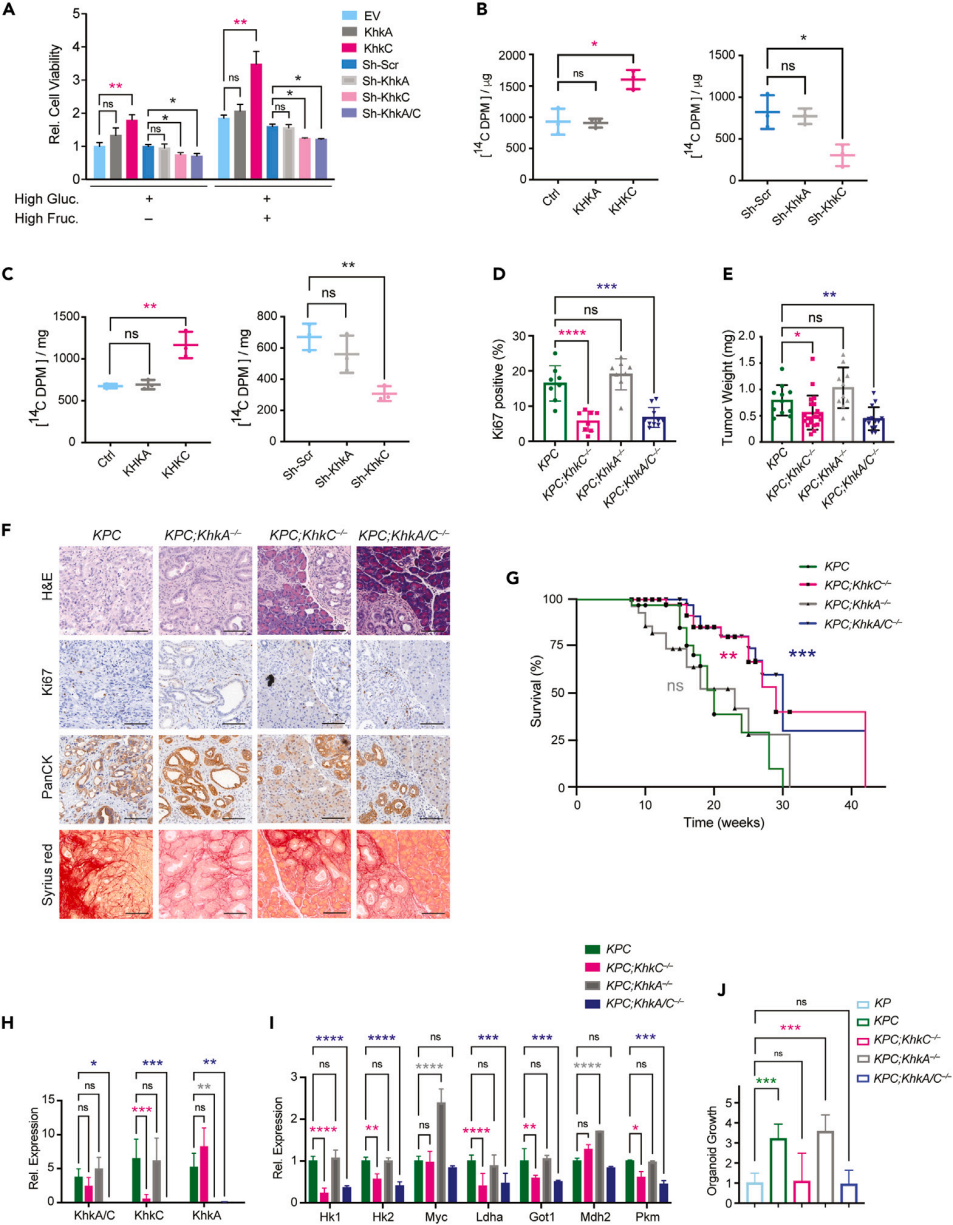
KHKC overexpression promotes tumor growth and *KhkC* KO is sufficient to delay the onset and development of tumors (A) KhkA or KhkC was either over-expressed or downregulated by shRNAs in *KPC* organoids and cell viability was measured after a 48-h incubation in media with or without fructose, n = 3. ANOVA multiple comparison test (Tukey’s test). (B) [^14^C] fructose incorporation into DNA in *KPC* organoids overexpressing empty vector (Ctrl), KhkA or KhkC, or after knockdown using sh-Scr (Scramble control) sh-KhkA or sh-KhkC, respectively after a 16 h incubation, n = 3. (C) [^14^C] fructose incorporation measurements into protein of *KPC* organoids overexpressing empty vector (Ctrl), KhkA or KhkC or after knockdown using sh-Scr (Scramble control) sh-KhkA or sh-KhkC, respectively, following a 16 h incubation, n = 3. (D) Percentage of Ki67 positivity in mouse tumor sections with the indicated genotype, n = 34 in total. ANOVA multiple comparison test (Tukey’s test). (E) Evaluation of pancreas tumor weight from mice with the indicated genotypes at 20 weeks of age, n = 57. ANOVA multiple comparison test (Tukey’s test). (F) Representative IHC images of H&E, Ki67, PanCK and Sirius red staining from *KPC*, *KPC;KhkC*^*−/−*^; *KPC;KhkA*^*−/−*^; *KPC;KhkA/C*^*−/−*^ tumors at 20 weeks of age. (G) Percentage of survival of *KPC*, *KPC;KhkC*^*−/−*^; *KPC;KhkA*^*−/−*^; *KPC;KhkA/C*^*−/−*^ mice, n = 149. Kaplan-Meier survival curves were compared by Mantel-Cox log rank test. (H) Relative transcript levels of KhkA/C, KhkC, KhkA from mouse organoids derived from the indicated genotypes. (I) Relative transcript levels of metabolism genes from organoids of all four aforementioned genotypes. (J) Growth measurements of organoids from all indicated genotypes cultured for 4 days in 17.3 mM glucose, n = 6. ANOVA multiple comparisons test (Tukey’s test). Data are represented as Mean ± SEM. The p values were determined by Student’s *t* test (unpaired two-tailed) or ANOVA multiple comparison test (Tukey’s test) when mentioned, n.s. (non-significant), ∗p < 0.05, ∗∗p < 0.01 and ∗∗∗p < 0.001, ∗∗∗∗p < 0.0001.

To study the relevance of the different KHK isoforms in our PDAC model systems, we first genetically manipulated *KPC*-derived mouse cells with stable KhkC or KhkA overexpression or shRNA knockdown of KhkC and KhkA isoforms, respectively (Figures S3A and S3B). Interestingly, KHKC overexpression increased the cell viability of *KPC* cells, an observation that was even more profound upon high fructose treatment for 48h. On the contrary, KhkC knockdown reduced the cell viability of *KPC* cells to similar extent as the complete KhkA/C knockdown. KhkA overexpression or knockdown did not have any significant impact of *KPC* viability (Figure 3A). When *KPC* cells overexpressing the KhkC isoform were treated with [^14^C (U)] D-fructose in the medium they displayed higher ^14^C incorporation into protein and DNA compared to KhkA overexpressing cells. On the contrary, knockdown of KhkC impaired ^14^C D-fructose incorporation into protein and DNA (Figures 3B and 3C). Of note, analysis of KhkC overexpressing cells revealed enhanced activation of the MAPK pathway as shown by increased phosphorylation of ERK (pERK^Thr202/Tyr204^) and higher levels of ALDOB, while no difference in pERK was measured compared to the control. However, gain of KHK-A function resulted in a strong downregulation of the RPS6 phosphorylation that is known to correlate with decreased cell proliferation (Figure S3A).^32^^,^^33^ To study the impact of KHK isoforms in PDAC onset and development *in vivo* we generated conditional mouse models with specific *KhkA* and *KhkC* ablation, by flanking exons 3a and 3c, respectively, with floxP sites (Figures S3C and S3D). Similar to the *KhkA/C* total knock out mice, *KhkC* and *KhkA* floxed mice crossed with *p48-Cre* were viable and did not show any major pancreatic phenotype compared to C57BL/6 wildtype (WT) or *KP* mice (not shown).^34^ The mutant alleles were then crossed to the aforementioned tumor models to generate homozygous Trp53 null mice (referred to as *KPC*^*fl/fl*^). The models were further validated by confirming the specific loss of exon 3a and 3c upon Cre recombination (Figure S3E). *KPC*^*fl/fl*^ mice developed highly aggressive invasive PDAC with a short life span and median survival of 8 weeks. Genetic deletion of *KhkC*, *KhkA* or *KhkA/C* in this model revealed similar tumor weights at 8 weeks and similar overall survival (Figures S3F and S3G). As previously shown, *KPC* mice driven by *Trp53*^*lox/+*^ developed advanced invasive PDAC tumors with a median survival of 24 weeks. Surprisingly *KhkC* and *KhkA/C* deletion in a model with *Trp*53 haploinsufficiency strongly reduced the tumor proliferation as shown by Ki67 stainings and tumor weight. In addition, these mice had a prolonged median survival of 28 weeks and 30 weeks, respectively, even under normal chow diet conditions (Figures 3D–3G).

We next generated organoids with *KhkC*, *KhkA*, and *KhkA/C* deletions from *KPC* mice. Gene expression analysis from mouse organoids confirmed the loss of respective alleles (Figure 3H). We measured marked decreases in the expression of Hk1, Hk2, Ldha, Got1 and Pkm2, all involved in glucose metabolism, in *KPC;KhkC*^*−/−*^ and *KPC;KhkA/C*^*−/−*^ organoids compared to control *KPC* organoids in normal growth condition without high fructose. While these transcripts were not altered in *KPC;KhkA*^*−/−*^ organoids, they markedly upregulated the expression of Mdh2 and Myc (Figure 3I). In line with the *in vivo* results, *KhkC* depletion was sufficient to impair the growth of *KPC* organoids *in vitro,* leading to a growth phenotype similar to *KP* and *KPC;KhkA/C*^*−/−*^ organoids. In contrast, no differences were measured in cell viability of *KPC;KhkA*^*−/−*^ compared to control *KPC* organoids (Figure 3J).

### Pancreatic ablation of *KhkC* strongly dampens the proliferation and migration of *KPC* driven tumors in a cell autonomous manner

**Figure 4:**
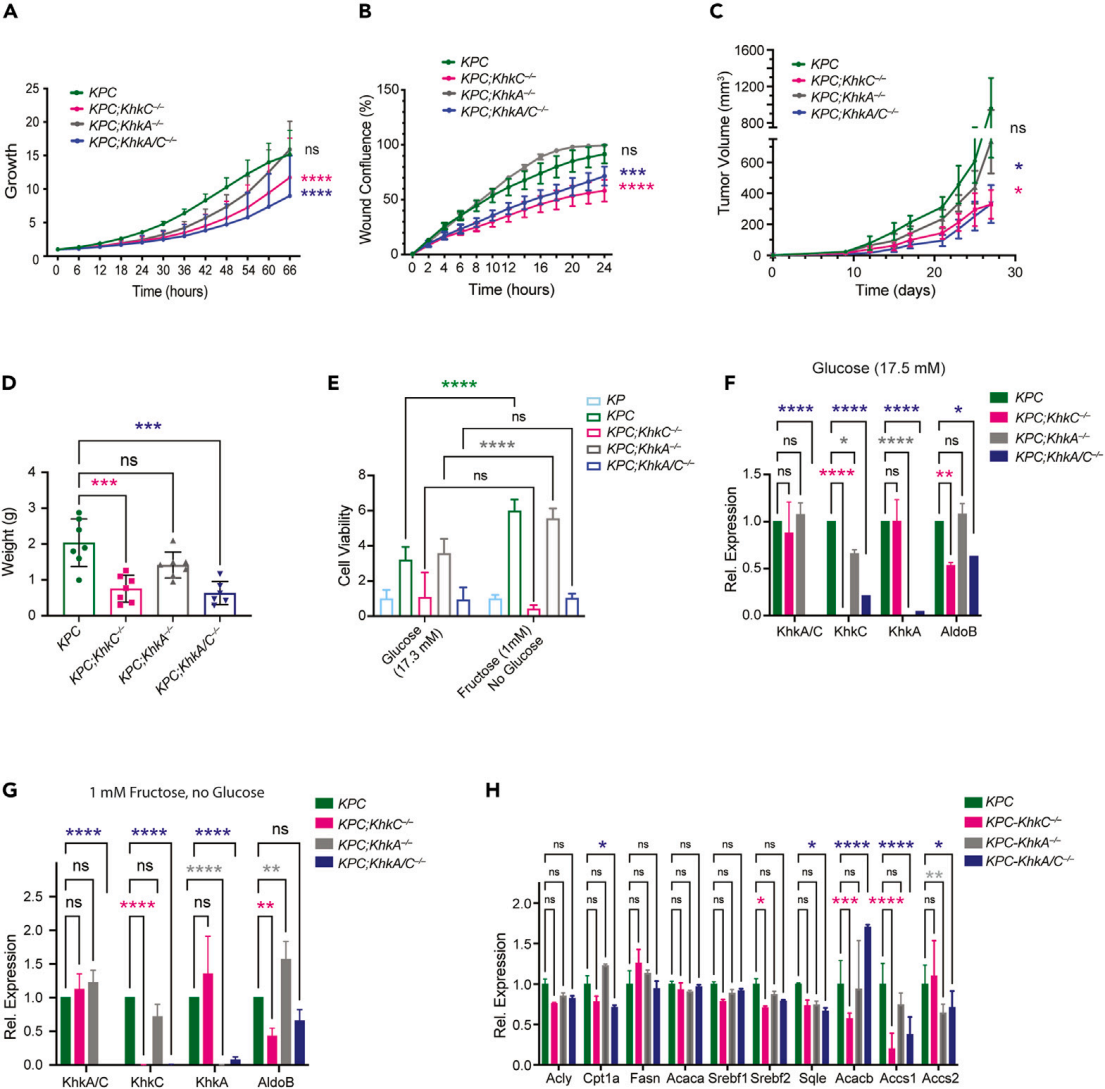
Pancreatic ablation of *KhkC* dampens the proliferation and migration of *KPC* driven tumors in a cell autonomous manner (A) Cell proliferation measurements of mouse derived cancer cells of *KPC*, *KPC;KhkC*^*−/−*^; *KPC;KhkA*^*−/−*^; *KPC;KhkA/C*^*−/−*^, determined by live-cell imaging analysis for 66 h, ANOVA multiple comparison test (Tukey’s test), n = 3. (B) Cell migration measurements of mouse derived *KPC*, *KPC;KhkC*^*−/−*^; *KPC;KhkA*^*−/−*^; *KPC;KhkA/C*^*−/−*^ cancer cells, determined by live-cell imaging analysis of wound healing confluence for 24 h. ANOVA multiple comparison test (Tukey’s test), n = 3. (C) Fold-change of the tumor volume (mm^3^) of *KPC*, *KPC;KhkC*^*−/−*^; *KPC;KhkA*^*−/−*^; *KPC;KhkA/C*^*−/−*^ cancer cells. ANOVA multiple comparison test (Tukey’s test), n = 8. (D) Tumor weight endpoints (in g) from *KPC*, *KPC;KhkC*^*−/−*^; *KPC;KhkA*^*−/−*^; *KPC;KhkA/C*^*−/−*^ xenograft tumors. ANOVA multiple comparison test (Tukey’s test), n = 7. (E) Cell viability measurements from *KPC*, *KPC;KhkC*^*−/−*^; *KPC;KhkA*^*−/−*^; *KPC;KhkA/C*^*−/−*^ mouse tumors organoids upon treatment with high glucose (17.3 mM) and high fructose (1 mM) for 4 days. (F) Relative transcript levels of KhkA/C, KhkC, KhkA and AldoB organoids treated with high glucose (17.3 mM). (G) Relative transcript levels of KhkA/C, KhkC, KhkA and AldoB from organoids of the indicated genotypes treated with high fructose (1 mM). (H) Gene expression analysis of fatty acid oxidation and lipid metabolism in high glucose diet from *KPC*, *KPC;KhkC*^*−/−*^, *KPC;KhkA*^*−/−*^, and *KPC;KhkA/C*^*−/−*^ organoids. ANOVA multiple comparison test (Tukey’s test), n = 3. Data are represented as Mean ± SEM. The p values were determined by Student’s *t* test (unpaired two-tailed) or ANOVA multiple comparison test (Tukey’s test) when mentioned, n.s. (non-significant), ∗p < 0.05, ∗∗p < 0.01 and ∗∗∗p < 0.001, ∗∗∗∗p < 0.0001.

To determine the role of KHK-C and its contribution to less aggressive PDAC tumor phenotypes, we sorted Epcam+ epithelial cells from mouse tumors of all four genotypes, followed by 2D-culturing and performing proliferation and cell migration assays. Similar to the 3D *in vitro* organoid models, *KPC;KhkC*^*−/−*^ and *KPC;KhkA/C*^*−/−*^ cells exhibited decreased growth rates compared to *KPC;KhkA*^*−/−*^ and *KPC* cells. In addition, by employing a scratch assay, we noted a significant decrease in the migratory capability of cells derived from *KPC;KhkC*^*−/−*^ and *KPC;KhkA/C*^*−/−*^ tumors (Figures 4A and 4B). These results were corroborated *in vivo* by employing PDAC xenograft models of all four genotypes, which showed a significant reduction in tumor volume and tumor weight from *KPC;KhkC*^*−/−*^ and *KPC;KhkA/C*^*−/−*^ compared to *KPC* or *KPC;KhkA*^*−/−*^ mice (Figures 4C and 4D). These results confirm that the specific inactivation of the KhkC isoform is sufficient to reduce the growth of PDAC xenograft tumors in a cell autonomous manner, even in absence of a high fructose diet.

*KPC* and *KPC;KhkA*^*−/−*^ organoids treated with a high-fructose diet showed enhanced cell viability compared to the organoids treated only with high glucose (Figure 4E). In high glucose conditions, quantitative RT-PCR analysis revealed reduced expression of AldoB in *KPC;KhkC*^*−/−*^ and *KPC;KhkA/C*^*−/−*^ organoids, but not in *KPC;KhkA*^*−/−*^ cells compared to KPC. In high-fructose conditions, AldoB levels were only significantly repressed in *KPC;KhkC*^*−/−*^ compared to *KPC* cells (Figures 4F and G), while AldoB levels were even higher expressed in *KPC;KhkA*^*−/−*^ compared to *KPC* organoids. This suggests that in the absence of *KhkC*, induction of AldoB is diminished due to decreased fructose metabolism, and conversely, lack of *KhkA* leads to increased AldoB expression, likely due to enhanced fructose metabolism. Interestingly, expression of genes involved in acetate metabolism and fatty acid synthesis, Acss1 and Acacb, were reduced upon deletion of *KhkC* or *KhkA/C*. Transcript levels of genes involved in cholesterol metabolism, including Srebf2 and Sqle were decreased only in *KPC;KhkC*^*−/−*^ and *KPC;KhkA/C*^*−/−*^, while transcripts of *de novo* lipogenesis showed similar expression levels, with the exception of the fatty acid oxidation gene Cpt1α, which was decreased upon inactivation of *KhkA/C* (Figure 4H). When challenging the mutant mice of the four aforementioned genotypes with a high fructose diet for 10 weeks and comparing them to chow diets, we measured increased proliferation and tumor weight only in *KPC* mouse tumors. Inhibition of proliferation was greater in all KHK-mutant genotypes under high fructose conditions, and even *KPC;KhkA*^*−/−*^ tumors showed no significant increase in Ki67 stainings compared to *KPC* control mice fed a high fructose diet, suggesting that under fructose stress both isoforms are required for tumor growth (Figures S4A–S4C). These effects could not be ascribed to changes in blood insulin and glucose, as there was no significant difference at 20 weeks of age (Figures S4D and S4E). Lastly, genetic inactivation of *KhkC* and *KhkA/C* profoundly reduced the overall survival of *KPC* mice even upon fructose diet, while *KhkA* ablation showed no significant difference (Figure S4F). These results demonstrat that dietary fructose enhances the proliferation of *KPC*-driven tumors *in vitro* and *in vivo* in a cell autonomous manner and that *KhkC* deletion is sufficient to reduce the growth and the migration of pancreatic cancer cells, leading to lower expression of fructose metabolizing genes downstream of KHK.

### Pancreatic *KhkC* inactivation rewires PDAC metabolism-related pathways

**Figure 5:**
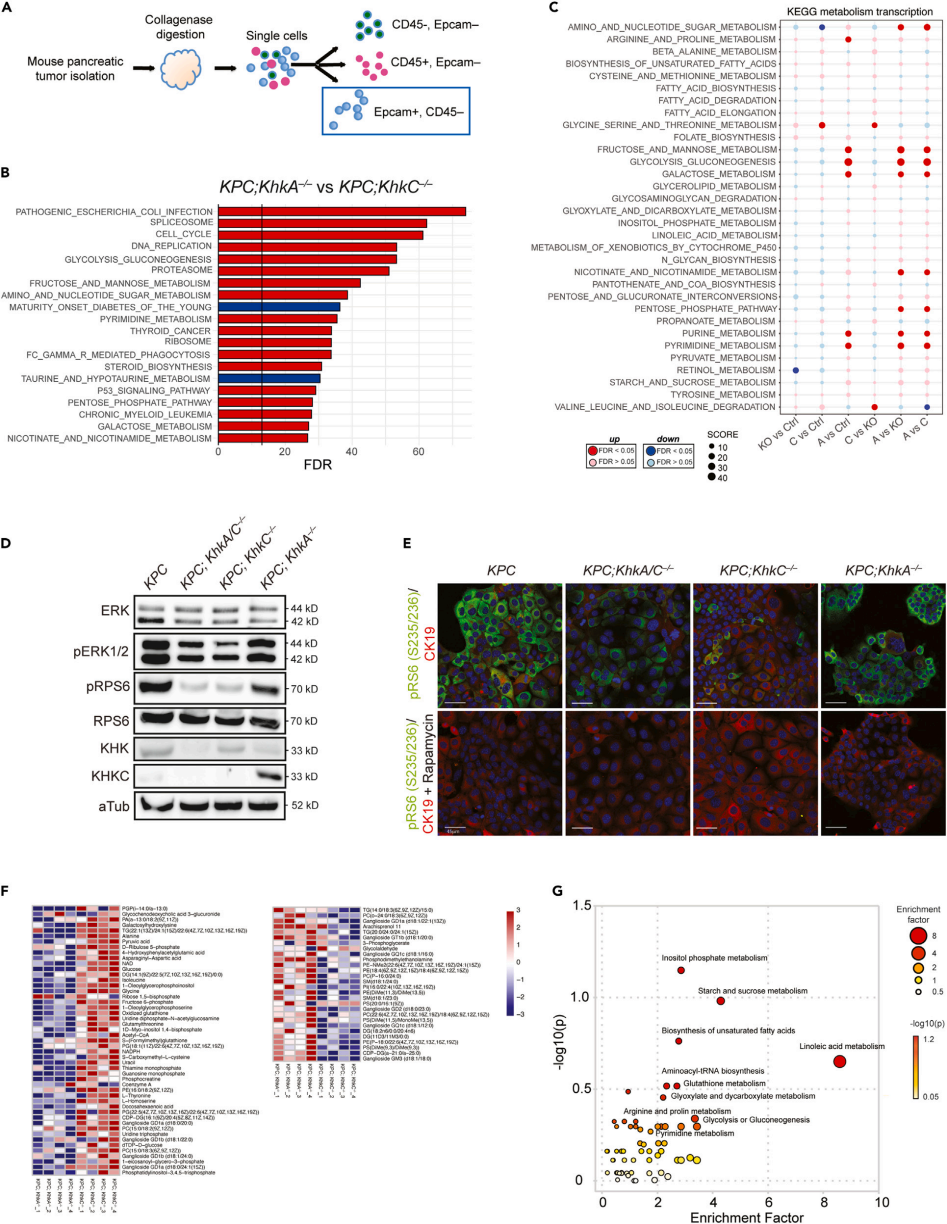
Pancreatic *KhkC* inactivation rewires PDAC metabolism-related pathways (A) Schematic of sorting strategy of Epcam+; CD45– cells from *KPC*, *KPC;KhkC*^*−/−*^; *KPC;KhkA*^*−/−*^; *KPC;KhkA/C*^*−/−*^ mouse tumors at 20 weeks of age. (B) Gene set enrichment analysis (GSEA) on the most regulated KEGG pathways from RNA-seq analysis of Epcam+; CD45– *KPC;KhkA*^*−/−*^ and *KPC;KhkC*^*−/−*^ mouse tumor cells (n = 2). p values are expressed in form of −10 × log 10FDR (FDR-adjusted). Upregulated: red; downregulated: blue. (C) Dot plots representing a KEGG analysis of metabolic pathways from *ex vivo* Epcam+; CD45^−^cells transcriptomics analysis. (*KPC*: n = 3; *KPC;KhkA/C*^*−/−*^: n = 3; *KPC;KhkC*^*−/−*^: n = 2; *KPC;KhkA*^*−/−*^: n = 2). p values are expressed in form of −10 × log 10 FDR (FDR-adjusted). Upregulated: red; downregulated: blue. (D) Representative Western blot of *KPC*, *KPC;KhkC*^*−/−*^, *KPC;KhkA*^*−/−*^, and *KPC;KhkA/C*^*−/−*^ mouse tumors cells showing the downregulation of *p*-ERK1/2 and *p*-RPS6. (E) Representative IF images from *KPC*, *KPC;KhkC*^*−/−*^, *KPC;KhkA*^*−/−*^, and *KPC;KhkA/C*^*−/−*^ cancer cells showing CK19 and phospho RpS6 expression (upper panel), and validation by treatment for 16 h with Rapamycin (lower panel). Scale bar: 45μm. (F) Heatmap showing the quantification of LC-MS metabolites of *KPC;KhkC*^*−/−*^ and *KPC;KhkA*^*−/−*^ cancer cell lines upon high fructose treatment for 48 h, n = 4. (G) Bubble chart of KEGG pathway enrichment. The size and color of each circle indicate the significance of the pathway ranked by p value (red: higher p values and yellow: lower p values) and enrichment factor (the larger the circle the higher the impact score), respectively.

To investigate the mechanisms by which KHK and its isoforms influence PDAC development, we performed RNA seq from sorted Epcam+; CD45– cancer cells of age-matched *KPC* mouse tumors and from tumors with genetic ablation of *KhkC, KhkA* and *KhkA/C* (Figures 5A and S5A). Hallmark gene set analyses from all four genotypes showed a significant reduction of cell cycle, inflammation, damage response, metabolism and cell signaling pathways, including Kras and mTORC1 signaling pathways, in tumors of both *KPC;KhkC*^*−/−*^ and *KPC; KhkA/C*^*−/−*^ mice compared to *KPC* and *KPC;KhkA*^*−/−*^ tumor cells (Figure S5B). Surprisingly, in the absence of a high fructose diet we measured higher expression levels of genes involved in pathways related to inflammation, damage response, cell cycle, metabolism, and mTorc1, Kras and Tgfβ signaling in *KPC;KhkA*^*−/−*^ compared to *KPC* alone, *KPC;KhkC*^*−/−*^ or *KPC;KhkA/C*^*−/−*^ tumor cells. Similarly, KEGG pathway analysis of *KPC;KhkA*^*−/−*^ versus *KPC;KhkC*^*−/−*^ confirmed a significant upregulation of pathways involved in cell cycle, DNA replication, pathways linked to fructose, mannose and galactose metabolism, pentose phosphate pathway, purine and pyrimidine metabolism, as well as nicotinate and nicotinamide metabolism, supporting a fundamental role for the *KhkC* isoform in key pathways involved in PDAC progression (Figures 5B and 5C).

Of note, analysis of organoids derived from *KPC;KhkC* and *KPC;KhkA/C* KO tumors confirmed a strong downregulation of the KRAS/MAPK pathway, as shown by lower expression of activated ERK1/2 (phospho-ERK1/2 Thr202/Tyr204) (Figure 5D). mTOR, a key downstream effector pathway of RAS^35^^,^^36^ involved in cell growth and protein synthesis,^35^^,^^36^ was strongly reduced in both *KPC;KhkC*^*−/−*^ and *KPC;KhkA/C*^*−/−*^ tumors, as shown by reduced expression of phospho-RPS6 in immunoblot analysis and by immunofluorescence staining of mouse tumor cells treated with mTOR inhibitor rapamycin, as well as from mouse tumor tissue sections (Figures 5D, 5E and S5C). Untargeted metabolomics from mouse tumor cell lines treated with low glucose (3 mM) and high fructose (1 mM) for 48 h confirmed numerous changes in metabolites between the *KPC;KhkC*^*−/−*^ and *KPC;KhkA*^*−/−*^. Specifically, analysis of *KPC;KhkC*^*−/−*.^showed an accumulation of glucose, fructose 6-phosphate, NAD, NADPH, phosphocreatine, ribulose 5-phosphate (an intermediate of the non-oxidative arm of PPP), pyruvic acid and acetyl-CoA, compared to *KPC;KhkA*^*−/−.*^cells (Figure 5F). In addition, glycine, isoleucine, L-homoserine levels were increased, while different species of lipids were downregulated in *KPC;KhkC*^*−/−*^ compared to *KPC;KhkA*^*−/−*^*,* indicating an inverse correlation between gene expression and metabolites, most likely due to their different metabolic consumption rate and growth. To perform metabolic pathway enrichment analysis, the human metabolome database (HMDB) ID’s of annotated metabolites from our input list were fed into the MetaboAnalyst database.^37^^,^^38^^,^^39^ A wide range of metabolic pathways were assigned as functionally enriched or over-represented, and between those, 11 were significantly enriched in *KPC;KhkC*^*−/−*^ and *KPC;KhkA*^*−/−*^ tumor cells, including inositol phosphate metabolism biosynthesis of unsaturated fatty acids, starch and sucrose metabolism, glyoxylate and dicarboxylate metabolism, glutathione metabolism, aminoacyl-tRNA biosynthesis, linoleic acid metabolism, glycolysis or gluconeogenesis, and pyrimidine metabolism (Figure 5G). Metabolic pathways that were predicted from RNA-seq data and related to metabolomic enrichments in cells from *KPC;KhkA*^*−/−*^ tumors confirmed an up regulation of interconnected biochemical reactions of glycolysis, gluconeogenesis and pyrimidine metabolism compared to *KPC;KhkC*^*−/−*^ tumor cells (Figure S5D). These data suggest that *KhkC KO,* and to similar extent *KhkA/C KO,* impairs Kras/MAPK and mTORC pathway gene expression and the activation of ERK1/2 and RPS6, rewires the expression of PDAC-related metabolism and signaling pathways, thereby impairing tumor progression.

## Discussion

Radical surgical resection combined with perioperative chemotherapy remains the only potentially curative option for PDAC patients. However, the recurrence rate is high, underscoring the need for better adjuvant therapies to improve long-term survival. Transformed cells rewire their metabolism to support tumor initiation and progression and often rely on critical nutrient sources. Therefore, exploiting the reprogrammed cancer metabolism provides an innovative therapeutic strategy. Epidemiological studies suggest that fructose consumption confers a greater pancreatic cancer risk than other sugars.^40^^,^^41^^,^^42^

KHKC is produced primarily in the liver, intestine, and kidneys, which efficiently metabolizes fructose and fuel gluconeogenesis, glycogen, triglyceride, and purine synthesis.^19^ In line with this observation are several human and rodent studies showing a correlation of fructose consumption with tumor growth in these tissues and profound effects on tumor growth when fructose metabolism is impaired.^24^^,^^26^^,^^27^^,^^42^^,^^43^^,^^44^^,^^45^^,^^46^^,^^47^ Increased fructose uptake contributes to enhance cellular survival and proliferation in many cancers.^12^^,^^23^ However, the function of specific KHK isoforms during cancerogenesis is still poorly understood. In cancers such as liver and breast cancer, both KHK isoforms have been shown to have distinct roles.^25^^,^^26^^,^^48^ In HCC, fructose metabolism appears to be inhibitory and is reduced in HCC cells through a mechanism involving c-myc-dependent transcriptional activation of hnRNP H1/2, which mediates a switch of the KHKC to the KHKA isoform.^16^ Using the *MUP-uPA* mouse model of NASH-driven HCC, a recent report demonstrated that consumption of a high-fructose diet promoted HCC development through a mechanism involving ER-stress-dependent barrier deterioration in the colon, endotoxemia and deterioration of steatohepatitis through activation of TLR signaling in liver macrophages.^17^ In breast cancer, overexpression of the KHKA isoform induced metastasis through 14-3-3 phosphorylation and recruitment of SLUG to the Cadherin 1 (*CDH1*) promoter under fructose-fed conditions, suggesting that KHKA, rather than KHKC, is necessary and sufficient for fructose-induced cell invasion.^48^

The impact of fructose metabolism in tumors derived from tissues with low KHKC expression is unknown. We and others have shown that KHK expression in the pancreas is markedly lower compared to liver and kidneys in normal pancreas, blurring its physiological relevance in physiological and malignant conditions. Whether dietary fructose effects are indirect or could also be explained by cell autonomous fructose metabolizing mechanisms is unknown, as no reports have yet addressed the specific role of KHK and its isoforms in PDAC development or in other cancerous tissues derived from organs with low KHK activity. Here, we show that KHK expression is induced in human PDAC tissue sections and in mouse *KPC* tumors that fully recapitulate pancreatic cancer. Furthermore, we demonstrate that fructose metabolism-related genes are overexpressed together with *Khk* in *KPC* tumor compared to the *KP* mouse organoids. *Khk* and the fructose metabolism-related gene expression are also enhanced upon hypoxia, a key feature of PDAC development.^49^ Moreover we show that high dietary fructose enhances the proliferation and decreases the overall survival due to the increase of KHK activity and higher expression of KHKC. Indeed, overexpression of the KhkC isoform in mouse *KPC* cells induces higher cell viability *in vitro* that results in even more viability upon high fructose treatment compared to KhkA overexpression. Using newly developed conditional mouse models of KhkC or KhkA deletion in the pancreas, we demonstrate that *KhkC* inactivation is sufficient to delay the onset and the development of PDAC, and significantly enhances the overall survival of *KPC*-driven tumor models, even in absence of high dietary fructose. These results emphasize an important role of KHK activity in PDAC despite its low expression in the normal pancreas.

Our study also revealed that loss of KHKC function decreases the viability of *KPC* organoids and cancer cells, the migratory capability of PDAC cells *in vitro*, and the growth of *KPC* cells *in vivo* in a cell autonomous manner when injected in immunodeficient mice. It is likely that these effects are mediated at least in part by the impaired activation of KRAS-MAPK pathway upon inactivation of KHKC or all KHK isoforms, as shown by the downregulation of phospho-ERK1/2 and by a decreased activation of RPS6, a downstream target of mTORC pathway. Conversely, we demonstrate that overexpression of KHKC in *KPC* cells enhances KRAS downstream pathway, while KHKA overexpression acts as a tumor suppressor by blocking the phosphorylation of ERK1/2 and decreasing RPS6 activation. In line with these findings, we found that transcriptomics data of Epcam+ CD45− sorted *ex vivo* cancer cells confirmed the regulation of mTORC1 and KRAS pathways as well as EMT and MYC targets genes, which were impaired in *KPC;KhkC*^*−/−*^ compared to *KPC;KhkA*^*−/−*^ or *KPC* tumor cells. Interestingly *KPC;KhkA*^*−/−*^ transcriptomics data revealed an opposite regulation of the transcription of genes compared to *KPC;KhkC*^*−/−*^ and to *KPC* involved in cell cycle and growth and more importantly in cell metabolism such as fructose and mannose metabolism, glycolysis, galactose metabolism and purine and pyrimidine metabolism, which could be accounted for enhanced fructose metabolism through KHKC overexpression. Metabolomics data analysis on cancer cells derived from our mouse models and treated with high fructose highlight an accumulation of many metabolites involved in glycolysis, pentose phosphate pathway in *KPC;KhkC*^*−/−*^ compared to *KPC;KhkA*^*−/−*^*,* suggesting altered consumption rates of these cells linked to their growth. Recent reports suggest that knocking down fructose metabolism by deleting KHK suppresses cancer growth in response to HFCS in APC mice.^24^ Furthermore, PF-06835919, a KHK inhibitor was developed and tested in a phase 2 clinical trial for NAFLD.^50^ The compound resulted in pronounced fructosuria, and reduced intrahepatic lipid in adults with NAFLD.^51^ Other reports have also shown that a ketogenic diet (low carbohydrates, low protein, high fat) results in increased intracellular NADH, priming PDAC cells for treatment with chemotherapy and leading to cytotoxicity and tumor regression.^52^ Here, we unraveled a potential role of KHK in early PDAC development and progression and propose KHK as a therapeutic target for adjuvant treatment of PDAC progression.

In summary, our findings show that increased KHKC expression in PDAC promotes its development and progression and that genetic inactivation of the KHKC isoform impairs fundamental pathways for PDAC growth and progression by impacting on key signaling and metabolic pathways, including KRAS-MAPK and mTORC signaling as well as glycolysis, pentose phosphate pathway, and pyrimidine/purine metabolism. Given the fact that existing inhibitors targeting KHK have been tested in clinical trials for nonalcoholic steatohepatitis (NASH), it would be of interest to analyze their effects as adjuvant therapies on PDAC patients. Together, our findings suggest that therapeutic targeting of KHK and fructose metabolism may be a strategy for slowing the progression of PDAC even in the absence of high dietary fructose intake.

### Limitations of the study

We point out that we have not been able to show increased expression of KHKC protein in biopsies of human pancreatic tumors because we did not have access toies suitable antibod for immunohistochemical stainings. Furthermore, it would be important to investigate the role of KHKA and KHKC isoforms in cancer cell lines derived from pancreatic cancers. In addition, organoids from human PDAC with defined driver genes, in combination with CRISPR-based approaches to obtain genetic knock-outs of KHK-specific isoforms, will be a powerful system to study the role of KHK in human PDAC and validate our findings in the mouse.

## STAR★Methods

### Key resources table

**Table:** 

REAGENT or RESOURCE	SOURCE	IDENTIFIER
**Antibodies**		

CD326 (EpCAM) Monoclonal Antibody (G8.8), FITC, eBioscience™	Invitrogen	Cat# 11-5791-82; RRID: AB_11151709
APC anti-mouse CD45 Antibody	Biolegend	Cat# 103112; RRID: AB_312977
Anti-mouse CD16/32 Antibody	Biolegend	Cat# 101302; RRID: AB_312801
Goat Anti-Mouse IgG Antibody (H+L), Biotinylated	Vector Laboratories	Cat# BP-9200; RRID: AB_2336171
Goat Anti-Rabbit IgG Antibody (H+L), Biotinylated	Vector Laboratories	Cat# BP-9100
HSP90 (C45G5) Rabbit mAb	Cell Signaling Technology	Cat# 4877; RRID: AB_2233307
Ki-67, Rabbit Monoclonal Antibody	Abcam	Cat# ab16667; RRID: AB_302459
Polyclonal Rabbit Anti-Cytokeratin, Wide Spectrum	Abcam	Cat# ab9377; RRID: AB_307222
TROMA III Cytokeratin 19	TROMA-III was deposited to the DSHB by Kemler, R. (DSHB Hybridoma Product TROMA-III)	Cat# ab-2133570; RRID: AB_2133570
Anti-Mouse IgG (H+L), HRP Conjugate	Sigma	Cat# 401253; RRID: AB_437779
Anti-Rabbit IgG (H+L), HRP Conjugate	Sigma	Cat# 401393; RRID: AB_437797
Goat anti-Mouse IgG (H+L) Cross-Adsorbed Secondary Antibody, Alexa Fluor 647	Invitrogen	Cat# A32728; RRID: AB_2633277
Goat anti-Rabbit IgG (H+L) Cross-Adsorbed Secondary Antibody, Alexa Fluor 488	Invitrogen	Cat# A-11008; RRID: AB_143165
Monoclonal Rabbit Phospho-S6 Ribosomal Protein (Ser235/236) antibody	Cell signaling	Cat# 4858; RRID: AB_916156
Polyclonal Rabbit Anti-Phospho-p70 S6 Kinase (Thr389) antibody	Cell signaling	Cat# 9205; RRID: AB_330944
Monoclonal Rabbit pS6 Ribosomal Protein antibody	Cell signaling	Cat# 2221
Polyclonal Rabbit Anti -p70 S6 Kinase antibody	Cell signaling	Cat # 9202; RRID: AB_331676
Monoclonal Rabbit Phospho-Akt (Ser473) antibody	Cell signaling	Cat# 4060; RRID: AB_2315049
Monoclonal Rabbit AKT (pan) antibody	Cell signaling	Cat# 4691; RRID: AB_915783
Monoclonal Mouse Anti-β-Tubulin antibody	Cell signaling	Cat# 3873S; RRID: AB_1904178
Monoclonal Rabbit Anti-β-Actin	Cell Signaling	Cat# 4970; RRID: AB_2223172
Monoclonal anti-β-Actin Antibody produced in mouse	Sigma	Cat# A2228; RRID: AB_476697
Monoclonal Mouse Anti KHK (B6)	Santa Cruz Biotecnology	Cat# sc-377411
Polyclonal Rabbit KHK C	SAB	Cat# 21709-2
Polyclonal Rabbit KHKA	SAB	Cat# 21708
Anti-KHK-A/C for immunohistochemistry	Sigma	Cat# HPA007040; RRID: AB_1079185
Monoclonal Rabbit Phospho-ERK (Thr202/Tyr204) antibody	Cell Signaling	Cat# 4370; RRID:AB_2315112
Monoclonal Rabbit ERK antibody	Cell Signaling	Cat# 4695s; RRID:AB_390779
Monoclonal Rabbit GLUT5 antibody	Santa Cruz Biotecnology	Cat# sc-271055
Monoclonal Rabbit ALDOB antibody	Abcam	Cat# ab153828

**Biological samples**		

Human Paraffin Tissues	Biobank University Hospital Heidelberg	N/A
Mouse PDAC tissues	This paper	N/A

**Chemicals and recombinant proteins**		

ProLong™ Gold Antifade Mountant with DAPI	Invitrogen	Cat# P36931
ImmPACT® DAB Substrate, Peroxidase (HRP)	Vector laboratories	Cat# SK-4105; RRDI: AB_2336520
Bovine Serum Albumin	Sigma-Aldrich	Cat#A2153
RIPA buffer (10X)	Cell Signaling Technology	Cat#9806
Phenylmethylsulfonyl Fluoride	Calbiochem	Cat# 329-9806
Mayer’s Hematoxylin	Diapath	Cat# C0303
30% Acrylamide/Bis solution 29:1	Bio-Rad	Cat# 1610156
Hoechst 33342	Thermo Fischer Scientific	Cat# H3570
D-(+)-Glucose	Sigma	Cat# G8270
D-(−)-Fructose	Sigma	Cat# F0127
[14C (U)] D-fructose	Hartmann Analytic	Cat# ARC0116; RRID: SCR_015807
Sirius Red	Sigma	Cat# 36-554-8
TrypLE	Gibco	Cat# 12605-010
Collagenase from *Clostridium Hystoliticum* type V	Sigma	Cat# C9263
RNAlater Stabilization Solution	Thermo	Cat# AM7024
AdDMEM/F12	Life Technology	Cat# 12634-028
SILAC Advanced DMEM/F-12 Flex Media, no glucose	Thermo	Cat# A2494301
L-Arginine	Sigma	Cat# A5006
L-Lysine	Sigma	Cat# L5501
GlutaMAX Supplement	Life Technology	Cat# 35050-061
HEPES	Sigma	Cat# H4034
B27	Thermo	Cat# 17504044
N-2 Supplement	Life Technology	Cat# 17502-048
N-Acetyl-L-cysteine	Sigma	Cat# A9165
Nicotinamide	Sigma	Cat# N0636
Recovery Cell Culture Freezing Medium	Life Technology	Cat# 12648-010
TrypLE Select Enzyme	Life Technology	Cat# A12177-01
Matrigel	Corning	Cat# 356255
FGF10	Peprotech	Cat# 100-26
Recombinant Human EGF	Peprotech	Cat# AF-100-15
Gastrin I	Sigma	Cat# G9020
Recombinant Human EGF	Peprotech	Cat# AF-100-15
Recombinant Murine Noggin	Peprotech	Cat# 250-38
RSPO-1	Peprotech	Cat# 120-38
Collagenase IV	Sigma	Cat# C5138
DNase I Solution	Thermo Scientific	Cat# 90083
HEPES (1M)	Gibco	Cat# 15630056
EdU (5-ethynyl-2'-deoxyuridine)	Life Technologies	Cat# A10044

**Critical commercial assays**		

High-Capacity cDNA Reverse Transcription Kit	Thermofisher	Cat# 4368813
CellTiter-Glo® 3D Cell Viability Assay	Promega	Cat# G9681
Insulin ELISA	ALPCO	Cat# 80-INSRTU-E10-AL
PicoPure® RNA Isolation Kit	Invitrogen	Cat# KIT0204
KAPA SYBR FAST ABI	Roche	Cat# SFABIKB
Gateway LR Clonase II enzyme mix	Invitrogen	Cat# 11791-100
Gateway BP Clonase II enzyme mix	Invitrogen	Cat# 11789-020
EnzyChrom Fructose Assay Kit	Medibena	Cat# BA_EFRU-100
Rneasy Mini Kit	Qiagen	Cat# 74104
Nucleospin Tissue	MACHEREY-NAGEL	Cat# 740952.50
Click-iT Plus Alexa Fluor 647 Picolyl Azide Toolkit	Life Technology	Cat# C1064
jetPRIME®	Polyplus transfection	Cat# 114-07/712-60

**Experimental models: Cell lines**		

HEK293T	ATCC	Cat# CRL-11268
Human organoid lines	This paper	N/A
Mouse organoid lines	This paper	N/A
Mouse PDAC cell lines	This paper	N/A

**Experimental models: Organisms/strains**		

Ptf1atm1(cre)Hnak/RschJ	The Jackson Laboratory	RRID:IMSR_JAX:023329
B6.129P2-Trp53tm1Brn/J	The Jackson Laboratory	RRID:IMSR_JAX:008462
B6.129S4-Krastm4Tyj/J	The Jackson Laboratory	RRID:IMSR_JAX:008179
LSL-Kras^G12D/+^; LSL-Trp53^R172H/+^	Tyler Jacks Lab	N/A
LSL-Kras^G12D/+^; LSL-Trp53^R172H/+^; Ptf1a-Cre (KPC^mut^)	This paper	N/A
C57BL/6J-KhKCtm1	Ozgene/ This Paper	N/A
C57BL/6J-KhKAtm1	Ozgene/ This Paper	N/A
KPC: LSL-KrasG12D/+;LSL-Trp53fl/+;Ptf1a-Cre	This paper	N/A
KPC-Khk-A^-/-^: LSL-KrasG12D/+;LSL-Trp53fl/+;Ptf1a-Cre-Khk-A ^-/-^	This paper	N/A
KPC-Khk-C^-/-^: LSL-KrasG12D/+;LSL-Trp53fl/+;Ptf1a-Cre-Khk-C ^-/-^	This paper	N/A
KPC-Khk-A/C^-/-^: LSL-KrasG12D/+;LSL-Trp53fl/+;Ptf1a-Cre-Khk-A/C^-/-^	This paper	N/A
Ptf1a-Cre-Khk-A^-/-^	This paper	N/A
Ptf1a-Cre-Khk-C ^-/-^	This paper	N/A
Ptf1a-Cre-Khk-A/C^-/-^	This paper	N/A
Khk-A/C^-/-^	R. Johnson (University of Colorado)	N/A

**Deposited data**		

RNA-seq	This paper	

**Oligonucleotides**		

Sh-RNA KhkA-1 Fwd	This paper, see Table S1	N/A
Sh-RNA KhkA-1 Rev	This paper, see Table S1	N/A
Sh-RNA KhkA-2 Fwd	This paper, see Table S1	N/A
Sh-RNA KhkA-2 Rev	This paper, see Table S1	N/A
Sh-RNA KhkC-1 Fwd	This paper, see Table S1	N/A
Sh-RNA KhkC-1 Rev	This paper, see Table S1	N/A
Sh-RNA KhkC-2 Fwd	This paper, see Table S1	N/A
Sh-RNA KhkC-2 Rev	This paper, see Table S1	N/A

**Oligonucleotides**		

*Hif1*α _Fwd	This paper, see Table S1	N/A
*Hif1*α _Rev	This paper, see Table S1	N/A
*KhkA/C*_Fwd	This paper, see Table S1	N/A
*KhkA/C*_Rev	This paper, see Table S1	N/A
*Khk-A*_Fwd	This paper, see Table S1	N/A
*Khk-A* _Rev	This paper, see Table S1	N/A
*Khk-C*_Fwd	This paper, see Table S1	N/A
*Khk-C*_Rev	This paper, see Table S1	N/A
*Slc2a1*_Fwd	This paper, see Table S1	N/A
*Slc2a1*_Rev	This paper, see Table S1	N/A
*Hk2*_Fwd	This paper, see Table S1	N/A
*Hk2*_Rev	This paper, see Table S1	N/A
*Aldob*_Fwd	This paper, see Table S1	N/A
*Aldob* _Rev	This paper, see Table S1	N/A
*Slc2a5* _Fwd	This paper, see Table S1	N/A
*Slc2a5*_Rev	This paper, see Table S1	N/A
*β-actin_*Fwd	This paper, see Table S1	N/A
*β-actin_*Rev	This paper, see Table S1	N/A
*Pdx1*_Fwd	This paper, see Table S1	N/A
*Pdx1*_Rev	This paper, see Table S1	N/A
*Ck19*_Fwd	This paper, see Table S1	N/A
*Ck19*_Rev	This paper, see Table S1	N/A
*Sox9* _Fwd	This paper, see Table S1	N/A
*Sox9*_Rev	This paper, see Table S1	N/A
*Hnf6*_Fwd	This paper, see Table S1	N/A
*Hnf6*_Rev	This paper, see Table S1	N/A
*Ptf1a*_Fwd	This paper, see Table S1	N/A
*Ptf1a* _Rev	This paper, see Table S1	N/A
*Cpa1*_Fwd	This paper, see Table S1	N/A
*Cpa1*_Rev	This paper, see Table S1	N/A
*Amy*_Fwd	This paper, see Table S1	N/A
*Amy*_Rev	This paper, see Table S1	N/A
*Ngn3*_Fwd	This paper, see Table S1	N/A
*Ngn3*_Rev	This paper, see Table S1	N/A
*Chga*_Fwd	This paper, see Table S1	N/A
*Chga*_Rev	This paper, see Table S1	N/A
*Ins2*_Fwd	This paper, see Table S1	N/A
*Ins2*_Rev	This paper, see Table S1	N/A
*Klf4*_Fwd	This paper, see Table S1	N/A
*Klf4*_ Rev	This paper, see Table S1	N/A
*Muc5ac*_Fwd	This paper, see Table S1	N/A
*Muc5ac*_Rev	This paper, see Table S1	N/A
*Muc6*_Fwd	This paper, see Table S1	N/A
*Muc6*_ Rev	This paper, see Table S1	N/A
*Tff1*_Fwd	This paper, see Table S1	N/A
*Tff1*_ Rev	This paper, see Table S1	N/A
*Slc2a2*_Fwd	This paper, see Table S1	N/A
*Slc2a2*_Rev	This paper, see Table S1	N/A
*Hk1*_Fwd	This paper, see Table S1	N/A
*Hk1*_Rev	This paper, see Table S1	N/A
*Myc*_Fwd	This paper, see Table S1	N/A
*Myc*_Rev	This paper, see Table S1	N/A
*Ldha*_Fwd	This paper, see Table S1	N/A
*Ldha*_Rev	This paper, see Table S1	N/A
*Got1*_Fwd	This paper, see Table S1	N/A
*Got1*_ Rev	This paper, see Table S1	N/A
*Mdh2*_Fwd	This paper, see Table S1	N/A
*Mdh2*_Rev	This paper, see Table S1	N/A
*Pkm_*Fwd	This paper, see Table S1	N/A
*Pkm_* Rev	This paper, see Table S1	N/A
*Acly*_Fwd	This paper, see Table S1	N/A
*Acly*_Rev	This paper, see Table S1	N/A
*Cpt1a*_Fwd	This paper, see Table S1	N/A
*Cpt1a*_Rev	This paper, see Table S1	N/A
*Fasn*_Fwd	This paper, see Table S1	N/A
*Fasn*_Rev	This paper, see Table S1	N/A
*Acaca*_Fwd	This paper, see Table S1	N/A
*Acaca*_Rev	This paper, see Table S1	N/A
*Srebf1*_Fwd	This paper, see Table S1	N/A
*Srebf1*_Rev	This paper, see Table S1	N/A
*Srebf2*_Fwd	This paper, see Table S1	N/A
*Srebf2*_Rev	This paper, see Table S1	N/A
*Sqle*_Fwd	This paper, see Table S1	N/A
*Sqle*_Rev	This paper, see Table S1	N/A
*Acacb*_Fwd	This paper, see Table S1	N/A
*Acacb*_Rev	This paper, see Table S1	N/A
*Accs1*_Fwd	This paper, see Table S1	N/A
*Accs1*_Rev	This paper, see Table S1	N/A
*Accs2_*Fwd	This paper, see Table S1	N/A
*Accs2_*Rev	This paper, see Table S1	N/A
*Cycloph*._Fwd	This paper, see Table S1	N/A
*Cycloph*._Rev	This paper, see Table S1	N/A
*18S_*Fwd	This paper, see Table S1	N/A
*18S*_Rev	This paper, see Table S1	N/A

**Recombinant DNA**		

pET11a KHK-C	Aruna Asipu (Leeds, GB)	N/A
pET11a KHK-A	Aruna Asipu (Leeds, GB)	N/A
psPAX2	Addgene	Cat# 12260; RRID: Addgene_12260
pMD2.G	Addgene	Cat# 12259; RRID: Addgene_12259
pLKO.1 - TRC Cloning Vector	Addgene Plasmid	Cat#10878; RRID: Addgene_10878

**Software and algorithms**		

Aperio ImageScope	Leica (V 12.3.2.8013)	https://www.leicabiosystems.com/digital-pathology/manage/aperio-imagescope/; RRID: SCR_020993
Image J	Schneider et al., 2012	https://imagej.nih.gov/ij/; RRID: SCR_003070
GraphPad Prism	GraphPad software (V 8.4.2 (679))	https://www.graphpad.com/scientific-software/prism/; RRID: SCR_002798

Cell Ranger (3.1.0)	NA	https://support.10xgenomics.com/single-cell-gene-expression/software/pipelines/latest/what-is-cell-ranger; RRID: SCR_017344
R v4.0.2	https://www.r-project.org/	NA
R package Seurat v3.2.0	https://satijalab.org/seurat/	RRID:SCR_016341
STAR v.2.5.1b	https://github.com/alexdobin/STAR	RRID:SCR_015899

### Resource availability

#### Lead contact

Further information and requests for resources and reagents should be directed and will be fulfilled by the lead contact, Markus Stoffel (stoffel@biol.ethz.ch).

#### Materials availability

Further information and requests for resources and reagents should be directed to and will be fulfilled by the lead contact, Markus Stoffel (stoffel@biol.ethz.ch).

### Experimental model and subject details

#### Mouse models

All mice were maintained under specific pathogen-free conditions a SPF animal facility at the ETH Phenomics Center (EPIC) at ETH Zürich. Maintenance and animal experiments were conducted in accordance with the Swiss Federal Veterinary Office (BVET) guidelines and approved by the local ethical committee, authorization numbers ZH018_15 and ZH055_17. *LSL-Kras*^*G12D*/+^ (Strain #:008179, LSL-K-ras G12D); *LSL-Trp53*^*fl/+*^ (Strain #008462, *p53*^*LoxP*^; Ptf1a-Cre (Strain #023329, p48-cre) were obtained from Jackson Laboratory and crossed to obtain *KPC*. *LSL-Kras*^*G12D/+*^; *LSL-Trp53*^*R172H*^ (*KP*), and *LSL-Kras*^*G12D/+*^; *LSL-Trp53*^*R172H/+*^; Ptf1a-Cre (*KPC*^*mut*^) were obtained from Tyler Jack’s (Howard Hughes Medical Institute). *KhkA/C*^*–/–*^ mice were obtained from R. Johnson (University of Colorado). *KhkC*^*flox/flox*^ and *KhkA*^*flox/flox*^ were generated by Ozgene Pty Ltd (Bentley WA, Australia) using goGermline technology. Briefly, A 499bp region (chr5:31,084,234-31,084,732 mm39) encompassing exon 3c was flanked with loxP sites for *KhkC*, while a 384 bp region (chr5:31,083,850-31,084,233 mm39) encompassing exon 3a was flanked with loxP sites. The genomic 5’ and 3’ arms of homology and the floxed genomic region were generated by PCR amplification of C57BL/6 genomic DNA. The FRT-flanked neo cassette was inserted along with the 3' loxP site. Homologous recombination of the targeting vector was carried out by electroporation of C57BL/6 ES cell line and clones were selected for neomycin resistance. Correctly targeted ES clones were identified by Southern blot RFLP analysis and microinjected into goGermline blastocyts to generate germline chimeras.^51^ Male chimeric mice were obtained and crossed to C57BL/6J females to establish heterozygous germline offspring on pure C57BL/6 background. Following germline transmission, the *FRT-PGK-NeoR-FRT* cassette was deleted by mating to a transgenic line containing FLP recombinase. The *Flp* gene was removed by segregation in subsequent crosses. Pancreas-specific knockout of *KPC* transgenic mice were crossed with *KhkC*^*flox/flox*^ and *KhkA*^*flox/flox*^ mice to generate *KPC;KhkC*^*–/–*^ and *KPC;KhkA*^*–/–*^ mouse models. NSG mice were used for the xenograft PDAC models upon injection of pancreatic cancer cells derived from GEMMs. All mouse, except for the survival study, were euthanized at the age of 15 or 20 and 9 or 13 weeks for analysis.

#### Human sample collection

Paraffin-embedded pancreatic tissue from patients with PDAC and healthy controls were obtained from the Biobank (Pancobank) of the European Pancreas Center (EPZ), Department of General Surgery, Heidelberg University Hospital. All procedures were in compliance with the Ethics Committee of the medical faculty at the University of Heidelberg, Germany (Ethic vote No. S-708/2019) and the Helsinki Declaration. An informed consent was obtained from all patients. PDAC and donor samples were stained for KHK.

#### Pancreatic cancer cell culture

Cancer cells were derived from *KPC*, *KPC;KhkC*^*–/–*^, *KPC;KhkA*^*–/–*^, *KPC;KHKA/C*^*–/–*^ mouse tumors, sorted using Epcam+, CD45- strategy and were cultured in DMEM supplemented with 10% FBS and 1% penicillin/streptomycin (P/S). HEK-293T (human embryonic kidneys) cells were obtained from ATCC. Cells were cultured in DMEM supplemented with 10% FBS and 1% penicillin/streptomycin.

#### Human PDAC organoid culture

Human PDAC organoids were established from patients’ surgical specimens and cultured as described in full detail by.^53^ Human pancreatic tissue samples were provided by the Department of Pathology and Molecular Pathology, University Hospital Zurich, based on informed consent and study approval from the ethical committee (BASEC-Nr. 2017-01319). For all samples PDAC status was confirmed on corresponding tissue slides reviewed by board-certified pathologists. To establish organoid lines, tissue was chopped and digested in full medium containing collagenase type II (5 mg/ml). The digestion was stopped with advanced DMEM/F12 medium supplemented with HEPES (10 mM), Glutamax (1%) and P/S (1%). Cells were seeded as 20μl drop of Matrigel (Corning, growth factor reduced) into a 48-well suspension culture plate. Human PDAC organoids were cultured in Advanced DMEM supplemented with 1x10^−4^ M HEPES, 1x Glutamax, 1% Penicillin/Streptomycin, 1x B27, 1.25 x10^−3^ M N-acetylcysteine, 50% Wnt3a conditioned medium (CM), 10% R-spondin-1 CM, 10% noggin CM, 1x10^−4^ M nicotinamide, 1x10^−6^ M prostaglandin E2, 50 ng mL^−1^, EGF, 10 x 10^−9^ M gastrin, 100 ng mL^−1^ FGF10 and 0.5 x 10^−6^ M A83-01.

#### Murine PDAC organoid culture

Pancreatic ducts were isolated from the whole organ of 20 weeks old KP, *KPC*, *KPC;KhkC*^*–/–*^, *KPC;KhkA*^*–/–*^, *KPC;KhkA/C*^*–/–*^ mice as previously described.^54^ Each organoid line was isolated from an individual mouse. Isolated ducts organoids were embedded in growth-factor reduced (GFR)-Matrigel (Corning), and cultured in organoid medium (OM), which is composed of AdDMEM/F12 (Gibco) supplemented with GlutaMAX (Gibco), HEPES (Gibco), Penicillin-Streptomycin (Invitrogen), B27 (Gibco), N-2 (Gibco), 1.25 mM N-Acetyl-L-cysteine (Sigma), 10 nM Gastrin I (Sigma) and the growth factors: 100 ng/ml FGF10 (Peprotech), 50 ng/ml EGF (Peprotech), 100 ng/ml Noggin, 100 ng/ml RSPO-1 (Peprotech), and 10 mM Nicotinamide (Sigma). For the first week after duct isolation the culture medium was supplemented with 100 μg/ml Primocin (InvivoGen).

### Method details

#### Organoid proliferation assays

Single cells were obtained by re-suspending organoids in medium through a fire-polished glass pipette, and then by enzymatic dissociation with TrypLE (Life Technologies). Cells (1500 cells per well) were embedded into growth factorreduced Matrigel and seeded into a 96 well for incubation. Following 96 hours of culture, cell proliferation was determined by measuring cell viabilities (G9681, Promega) using the microplate reader (Tecan). Six replicate wells per condition were used. Luminescence data were analyzed with GraphPad Prism.

#### 2D cell proliferation assay

For the proliferation assay, 1x10^5^ cells/well were seeded in a 96-well plate and incubated for 72 h. Proliferation was monitored and analyzed by using Incucyte S3 *in vitro* system (Essenbioscience). Four pictures were recorded every 6 hrs for the 4 biological replicates and 6 technical replicates.

#### Migration assay

Migration assay was performed in 2D cancer cells derived from the four mouse genotypes by plating 3 × 10^5^ cells per well of 96-well plate. Wound was performed using Incucyte Wound Maker and cell migration was monitored and analyzed by using Incucyte S3 *in vitro* system (Essenbioscience).

#### Plasmid constructions of over-expression and shRNAs knockdown of KhkA, KhkC and KhkA/C

The KHKA and KHKC overexpression plasmids were produced by sub cloning KHKA and KHKC from the pET11a-KHKA and pET11a-KHKC expression constructs (provided by A. Asipu) into the Gateway Entry vector pENTR1a and by LR-reaction in the pLenti-pgk-puro lentiviral expression vector. siRNAs targeting KhkA, KhkC or KhkA/C were designed using the Life Technologies BLOCK-iT RNAi Designer online tool (Life Technologies), searching for targets to KhkA, KhkC, KhkA/C transcripts. The TRC loop sequence CTCGAG was used. Two shRNAs were chosen as candidates for each isoform and cloned into the pLKO.1 lentiviral vector respectively. The presence of the shRNA was confirmed by sequencing (Microsynth). ShRNAs were then tested in organoids for knockdown efficiency and specificity. Sh-KhkC, Sh-KhkA, Sh-KhkA/C sequences used in this study are listed in the Key Resource Table and Table S1. A non-targeting shRNA vector SHC002 (Sigma) was used as a control.

#### Lentivirus production and transduction

Lentiviral particles were produced in HEK-293T cells that were purchased from American Type Culture Collection (ATCC). To prepare lentiviral particles, HEK-293T cells were transfected using JetPRIME® transfection reagents (JetPRIME®, Polyplus transfection, 114-07/712-60) as per the manufacturer’s instructions. The virus in the supernatants from a 10 cm dish was concentrated by ultracentrifugation at 60,000g for 2 hrs at 4°C (Beckman). The viral concentrate was re-suspended in 50 μl PBS. Following dissociation of the organoids, the fragments were transduced with lentivirus by mixing with virus-containing medium in a 48-well plate. The mixture was then centrifuged at 600g for 1 h at room temperature and incubated for 6 h at 37°C. After spin down, the pellet was re-suspended in matrigel (Corning) and seeded 40 μl into a 24-well plate. The plate was incubated at 37°C for 5 to 15 min until the basement matrix was solidified and then adding the culture medium. Selection was performed by adding puromycin (1 μg/ml) to the medium.

#### Insulin Elisa assay

To determine mouse serum insulin levels, blood was obtained from the tail vein and centrifuged for serum separation. Insulin was measured by Insulin ELISA Kit (ALPCO, 80-INSRTU-E10-AL) following the instructions.

#### Glucose measurement

Blood glucose values were measured with a Bayer Contour XT glucometer after 6 h of fasting at the endpoint of the treatment.

#### Immunohistochemistry (IHC) and immunofluorescence (IF)

For both IHC and IF tissue sections were processed as follows: deparaffinizing, unmasking, pre-staining, blocking and secondary stainings. Deparaffinization was performed using six-step procedure of alcohol scale 5mins each. The slides were drained off the excess solution and were then immersed in ionized water for 5 min. Further, unmasking or antigen retrieval procedure was followed which involved immersing the section slides in solution pH 6 (Citrate, Company: Diapath, Cat No. T0050) in the microwave at 98°C for 25 min. The slides were allowed to cool at room temperature for 25 min. The section slides were washed with 1xPBST (0.5% Tween20), twice for 3 min each, followed by staining procedure. Only for IHC but not for IF, blocking procedure began by incubating the slides with 3% H_2_O_2_ for 10 min, and followed by 1xPBST washes as before and performing protein block. For both IHC and IF, protein blocking was performed using Protein-Block solution (DAKO Agilent technologies, Cat No. X0909) for 10 min at room temperature. When antibodies were developed in mice, the tissues were blocked for mouse cross-reactivity using a biotinylated anti-mouse antibody (Vector Laboratories, Cat No. BP-9200). Sections were stained with respective primary antibodies at room temperature for 1 h followed by 3 washes with 1xPBST as before. These slides were further incubated with respective secondary antibodies, for IHC, anti-Mouse (Vector Laboratories, Cat No. BP-9200), anti-Rabbit (Vector Laboratories, Cat No. BP-9100) and for IF using aluminum foil to protect from light, Fluor chrome antibodies (conjugated with either with Alexa- 488, -647). In both cases, secondary antibodies were diluted at 1:200 in 1xPBS solution for 30 min at room temperature. For IF, upon completion of secondary antibody incubation procedure, slides were washed 3 times with 1xPBST with minimum exposure to light and upon draining out the PBST, slides were incubated with mounting media with DAPI (Invitrogen, P36931) ready to be visualized under a fluorescent confocal microscope. For IHC, during secondary antibody incubation, Vectastain ABC solution was prepared (Company: Vector laboratories, Cat No. PK-6100) at the dilution of 1:150 of both Solution A and Solution B in 1xPBS solution followed by 30 min incubation at room temperature. Upon completion of secondary antibody stainings, slides were washed for 3 times with 1xPBST followed by ABC solution staining for 30 min at room temperature. After ABC, slides were washed 3 times with 1xPBST and final steps of IHC stainings were performed. DAB staining was performed using DAB solution (Company: Vector laboratories, Cat No. SK-4105. One drop of Chromogen in 1ml of Diluent solution) and allowed to stain for no more than 2-3 min at room temperature. Immediately slides were washed 3 times with 1x PBST and counter staining was performed using hematoxylin solution (Diapath, C0303). At the end of IHC staining, sections were dehydrated using deparaffinization procedure after which slides were mounted with coverslip using mounting media (Sigma, 03989). The Sirius red staining was performed using a solution of Picro-sirius red (0.5 g) and saturated aqueous solution of picric acid (500 ml) in de-waxed and hydrated paraffin sections for 1 h followed by two washing steps with acidified water (5 ml acetic acid glacial to 1 liter of distilled water), the dehydrate in three changes of 100% ethanol, clear in xylene and mount.

Normal, tumor tissue samples were fixed in 10% neutral-buffered formalin overnight. Tissues were washed thoroughly under running tap water followed by processing using ethanol and embedded in paraffin according to standard protocols. Sections (3 μm) were prepared for antibody detection. Slides were prepared as consecutive sections. Human PDAC and donor section used was provided by the PancoBank of the European Pancreas Center Heidelberg and used in accordance with the regulations of the biobank and the vote of the ethics committee of the University of Heidelberg.

#### Ketohexokinase activity assay

The ketohexokinase activity assay was modified by adding Protease Inhibitor Cocktail (Roche) and PhosSTOP (Roche) to the reaction mixture.^19^ The reaction was performed at 37°C for 2 h after adding 140 μg protein for each reaction. The remaining fructose was measured using a Spectra MAX 190 plate reader (Molecular Devices). Reactions without protein were used as negative controls, and lysate from KHK-C overexpression cells was used as a positive control.

##### Lysis

• Lysis Buffer (1 ml): 800uL water, 50 μl 1M Imidazol buffer pH 7, 150 μl KCl, 0.5 M
• Lyse 10 wells of organoids in 100 μl Lysis Buffer or 100 mg tissue in 1 ml lysis buffer.
• Sonication: 10 to 15 pulses, 40% amplitude
• Centrifugation: 15 min at 15,500 g, 4°C
• Determine protein concentration with BCA kit

##### Preparation of reaction mixture

• Prepare 0.1 M D-Fructose solution fresh (e.g. 180 μg Fructose in 10 mL water)
• For one reaction in a volume of 100 μl, add in this order:
• 0.9 μl 0.1 M D-Fructose
• 8 μl 0.5 M NaATP
• 0.8 μl MgCl_2_
• 20 μl 5 M KAc
• Mix by pipetting up and down several times
• 8 μl 0.5 M NaF
• 10 μl 0.2 M N-Acetylglucosamine
• 5 μl 1 M Imidazole Buffer pH 7
• up to μl 100 H_2_O
• Include T0 samples without protein (or with protein but without ATP and Mg)
and a blank (no fructose and ATP)

##### Reaction

• Add 140 μg protein to reaction mixture
• Incubate 2 h at 37°C in a heating block
• Stop reaction by the addition of 50 μl ZnSO_4_ (1 M) and 250 μl Ba(OH)_2_ (0.1 M)
• Centrifuge 15 min at 5500 g
• Take off supernatant

##### Seliwanoff reaction

• Use 100 μl of the supernatant
• Add 100 μl 0.1% alcoholic resorcinol
• Add 300 μl 30% HCl
• 8 mins at 80°C in a water bath at 37°C
• Let cool down at RT
• Measure duplicates in a 96 well plate (200 μl/well): absorbance at 515 nm.

#### [14C (U)] D-fructose incorporation into DNA and protein *in vitro*

Three lines of organoids for each condition were seeded in a 24-well plate and cultured in the complete medium. After 2 days, fresh medium containing 1 μCi/ml [^14^C (U)] D-fructose (Hartmann Analytic) was added and incubated overnight. Then, organoids were washed three times with PBS and proteins were extracted as described in Western blot section. Genomic DNA was extracted using a kit according to manufacturers’ instructions (MACHEREY-NAGEL, 740952.50). Radioactivity was measured in a Beckman LS6500 scintillation counter (Beckman) and normalized to the amount of DNA or protein.

#### Western blot

Mouse pancreatic tumor samples or organoids were lysed using 1x RIPA buffer (Cell signaling, 9806) supplemented with Phenylmethanesulfonyl fluoride (PMSF; Millipore Sigma, catalog 329-98-6) and incubated on ice for 20 min. Samples were centrifuged at 14’000 rpm for 20 min. Protein concentration was determined by the BCA kit (Thermo Fisher 23227). Equal amounts of protein were subjected to SDS-polyacrylamide gel electrophoresis (SDS-PAGE), 10% based on the molecular weight of the proteins of interest and transferred on to 0.45 μm nitrocellulose membrane (Perkin Elmer, NBA083C001EA). After protein transfer, membranes were blocked in 5% milk solution and membranes were probed with the indicated antibodies overnight at 4°C. The membranes were incubated with horseradish peroxidase-conjugated (HRP-linked) secondary antibodies anti-rabbit IgG (Sigma, 401393, 1:5000) or anti-mouse IgG (Sigma, 401253, 1:5000) and developed using enhanced chemoluminescence (ECL) substrate. Membranes were exposed to Fusion Solo S imaging system (Vilber). Blots were semi-quantitatively analyzed by densitometry using ImageJ 1.52 v (National Institutes of Health). Primary antibody against were used at the following concentration: KHKA/C (1:500, Santa Cruz), KHKC (1:500, SAB), KHKA (1:500, SAB), p-ERK (1:1000, CST), ERK (1:1000, CST), p-AKT (1:1000), AKT (1:1000, CST), GLUT5 (1:500, Santa Cruz), Aldolase B (1:1000, Abcam), Phospho-p70 S6K (1:1000, CST), Phospho-S6 (Ser235/236) (1:1000, CST) p70 S6K (1:1000, CST), pS6 (1:1000, CST) .

#### Antibodies

Primary and secondary antibodies were used at the concentrations indicated in the Key Resources Table according to manufacturer’s instructions.

#### EdU incorporation assays

EdU incorporation-based proliferation assay was performed with *KPC* organoids. Briefly, 10 μM EdU (Life Technology, C10643) was added to the organoid cultures and pulsed for 4 h. Subsequently, organoids were recovered from matrigel and were dissociated into single cells by TrypLE (Gibco) and mechanical mean using a fire-polished glass Pasteur pipette. Cells were fixed with 4% paraformaldehyde (PFA) in PBS for 10 min at 4°C. PFA was removed and cells were twice washed with PBS. After permeabilizing the cells in PBS containing 0.2% Triton-X100 (PBS-T) for 15 min at 4°C, the “click-iTEdU” reaction was carried out using the Click-iT Plus EdU Alexa Fluor 647 Picolyl Azide Toolkit (Life Technology, C10643) according to the manufacturer’s instructions. Cells were then resuspended in 500 μl PBS and subjected to FACS analysis (BD LSRFortessa; BDFACSDiva software). EdU incorporation imaging was performed as described above without any dissociation of the organoids. The stained organoids were imaged under confocal microscopy (Leica).

#### Real-time PCR analysis

RNA from organoids was isolated with the RNeasy Mini Kit (Qiagen) and reverse-transcribed to cDNA (Applied Biosystems) according to the manufacturer’s instructions. RNA from pancreata was isolated by immediately immersing the dissected tissue into RNAlater and cutting into small pieces. After homogenized by freeze slamming wtih Trizol and centrifugation, the supernatant was transferred into a 2 ml tube followed by spinning with chloroform. The upper aqueous layer was used for the RNA extraction with the RNeasy Mini Kit. First-strand cDNA was synthesized with random hexamer primers using the High-Capacity RNA-to-cDNA Kit (No. 4368813; Applied Biosystems).

Quantitative real-time PCR reactions were carried out with 2x KAPA SYBR FAST qPCR Mastermix (No. KK4601; KAPA Biosystems) and run on a Light Cycler 480 (Roche). Ct values were normalized to the housekeeping genes beta-actin, 18S rRNA or cyclophilin (Ppia). The sequences of primers used in this study are listed in the Table S1. Expression levels were calculated using the 2^-ΔΔCT^ method^55^

#### Fructose or glucose feeding

25% of fructose (Sigma, F0127) was dissolved in tap water. The solution was filtered using a filter bottle (Milian SA, 99500). Fructose feeding experiments started when mice were at the age of 10 weeks. The water was refreshed every 5 days.

#### Analysis of metabolites in cancer cell lines

5x10^5^ cancer cells from all four mouse genotypes were plated in 10 cm dish in DMEM 10% FBS 3 mM glucose and 10 mM of fructose. After 48 h, the growth medium was removed from the plate and the cells were washed with AMBIC (ammonium bicarbonate) buffer at room temperature. 400 μl of 80% ice cold MeOH were added to the plate and cells were detached from surface by scraping gently. The procedure was repeated a second time, to complete the cell harvesting, and the cell suspensions obtained in the two harvesting rounds were pooled in a single sample. Sample homogenization and metabolite extraction has been achieved by freeze-thaw-cycle lysis. The tubes containing the cell suspension were placed in liquid N2 for 2 min, followed by 5 min incubation at room temperature in a shaker (600 rpm). Such cycle was repeated 4 times, followed by vortexing/mixing by pipetting and transfer a fixed volume of suspension to a new test tube. The metabolite enriched supernatant was separated from the cell debris by centrifugation at 10,000 g for 10 min at 4°C after a 15 min incubation of the cell suspension on ice. The pellet was kept for protein quantification and the results of such quantification were used to normalize the concentration of the metabolites extracts.

#### Sample preparation for LC-MS analysis and LC-MS analysis

100 mL methanol extracts were dried under nitrogen stream, reconstituted in 20 μl water (MS grade) plus injection buffer (90% ACN, 8.8% MeOH, 50 mM ammonium acetate, pH 7.0). The solution was vortexed and centrifuged at 16,000 x g, 4°C for 15 min. 50 ml of the supernatant were transferred to a glass vial with narrowed bottom (Total Recovery Vials, Waters) for LC-MS injection. In addition, method blanks, QC standards, and pooled samples were prepared in the same way to serve as quality control for the measurements.

Metabolites were separated on a nanoAcquity UPLC (Waters) equipped with a BEH Amide capillary column (150 mm x 130 mm, 1.7 mm particle size, Waters), applying a gradient of 5 mM ammonium acetate in water (A) and 5 mM ammonium acetate in 95% acetonitrile (B) from 90% B to 50% B over 10 min, followed by 2 min at 98% B and 4 min re-equilibration at 90% B. The injection volume was 1 ml. The flow rate was adjusted over the gradient from 3 to 2 ml/min. The UPLC was coupled to Synapt G2Si mass spectrometer (Waters) by a nanoESI source. MS1 (molecular ion) and MS2 (fragment) data were acquired using negative polarization and MS^E^ over a mass range of 50 to 1200 m/z at MS1 and MS2 resolution of >20,000.

#### Untargeted metabolomics data analysis

Metabolomics data sets were evaluated in an untargeted fashion with Progenesis QI software (Nonlinear Dynamics), which aligns the ion intensity maps based on a reference data set, followed by a peak picking on an aggregated ion intensity map. Detected ions were identified based on accurate mass, detected adduct patterns and isotope patterns by comparing with entries in the HMDB Data Base. A mass accuracy tolerance of 5 mDa was set for the searches. Fragmentation patterns were considered for the identifications of metabolites. All biological samples were analyzed at least in triplicate and quality controls were run on pooled samples and reference compound mixtures to determine technical accuracy and stability.

#### Illumina RNA sequencing

Pancreatic tumors from all four genotypes mouse models were resected and processed for single cell suspension followed by RNA sequencing and analysis using the following procedure:

-Single cell suspension: PDAC tumors were isolated, minced and processed for single cell suspension. Tissues were digested in 2 ml of Digestion Buffer composed by Collagenase IV 3% in RPMI, 10% FBS + 1% Pen/Strep, DNase I 30 U/μl, Hepes 10 mM. The cell suspension was incubated for 30 min at 37°C on a rocker. Then, the digestion was stopped adding 1 ml of RPMI 10% FBS + 1% P/S. The cells suspension was filtered through a 100 μm cell strainer and kept on ice for 4 min. Then cells suspension was filtered again through a 40 μm cell strainer and spun down at 1500 rpm for 5 min at 4°C. FACS staining was performed using the following antibodies: anti-Epcam-FITC and anti-CD45-APC and the Epcam+; CD45^–^ cells were sorted for total RNA extraction using PicoPure® RNA Isolation Kit (Invitrogen, KIT0204).

The quantity and quality of the isolated RNA was determined with a Fragment Analyzer (Agilent, Waldbronn, Germany). Due to the low RIN number of some samples the SMARTer Stranded Total RNA-Seq Kit - Pico Input Mammalian (Clontech Laboratories, Inc., A Takara Bio Company, California, USA) was used in the succeeding steps. Briefly, total RNA samples (0.25–10 ng) were reverse-transcribed using random priming into double-stranded cDNA in the presence of a template switch oligo (TSO). When the reverse transcriptase reaches the 5’ end of the RNA fragment, the enzyme’s terminal transferase activity adds non-templated nucleotides to the 3’ end of the cDNA. The TSO pairs with the added non-templated nucleotide, enabling the reverse transcriptase to continue replicating to the end of the oligonucleotide. This results in a cDNA fragment that contains sequences derived from the random priming oligo and the TSO. PCR amplification using primers binding to these sequences can now be performed. The PCR adds full-length Illumina adapters, including dual barcodes for multiplexing. Ribosomal cDNA is cleaved by ZapR in the presence of the mammalian-specific R-Probes. Remaining fragments are enriched with a second round of PCR amplification using primers designed to match Illumina adapters. The quality and quantity of the enriched libraries were validated using the Fragment Analyzer (Agilent, Waldbronn, Germany). The product is a smear with an average fragment size of approximately 360 bp. The libraries were normalized to 10 nM in Tris-Cl 10 mM, pH 8.5 with 0.1% Tween 20. The equimolar pool of samples was spiked into a NovaSeq6000 run targeting 200x10^6^ reads on a S1 FlowCell (Novaseq S1 Reagent Kit, 100 cycles, Illumina, Inc, California, USA).

#### RNA seq data analysis

The quality of the sequencing reads was assessed utilizing FastQC (version 0.11.9). Alignment of total RNA (stranded) to the reference mouse genome (M27) was carried out using STAR (version 2.7.9a) in a two-pass mode. Gene expression was quantified at the gene level through the use of comprehensive annotations provided by Gencode (vM27 GTF File). Samples were normalized for library size and transformed using the variance stabilizing transformation in R statistical environment with the DESeq2 (version 1.38.1) pipeline. In the differential expression analysis between groups, an Independent Filtering procedure was employed to exclude genes that were not significantly expressed in the majority of samples under consideration. Unless specified, all gene set enrichment analyses were performed using the Limma (version 3.54.0) package. Gene sets were obtained from the Molecular Signature Database (MSigDB) and Reactome. Multiple testing correction was applied to P-values using the false discovery rate (FDR) with a significance threshold of 0.05.

### Quantification and statistical analysis

All experiments were performed on biological replicates as mentioned in the respective figure legends. Sample size for each experimental group/condition is reported in the appropriate figure legends. All data points are presented for quantitative data, with an overlay of the mean with SEM. Statistically significant differences between control and experimental groups were determined using Multiple Student’s t-tests (two-tailed, unpaired), one-way ANOVA with Sidák’s and Tukey multiple comparison difference tests, and log-rank (Mantel-Cox) test as indicated in the appropriate figure legend and text. Significances are indicates as ∗P<0.05, ∗∗p<0.01 and ∗∗∗p<0.001, and ∗∗∗∗p<0.0001. All statistical analyses were performed using GraphPad Prism 9, Microsoft Excel 2016 or R-Studio.

## Data Availability

ArrayExpress RNA-seq accession number: ArrayExpress: E-MTAB-12746. No original code was generated in this study.
