# Supplementary material for: Genetic ablation of ketohexokinase C isoform impairs pancreatic cancer development
Source: iScience. 2023 Jul 13;26(8):107368. doi: 10.1016/j.isci.2023.107368 (PMC10407955; doi:10.1016/j.isci.2023.107368)
Supplement: Document S1. Figures S1–S5 and Table S1 [file mmc1.pdf]

iScience, Volume *26*

Supplemental information

Genetic ablation of ketohexokinase C isoform impairs pancreatic cancer development

Ilaria Guccini, Guanghui Tang, Trang Thuy To, Laura Di Rito, Solange Le Blanc, Oliver Strobel, Mariantonietta D'Ambrosio, Emiliano Pasquini, Marco Bolis, Pamuditha Silva, Hasan Ali Kabakci, Svenja Godbersen, Andrea Alimonti, Gerald Schwank, and Markus Stoffel

Figure S1

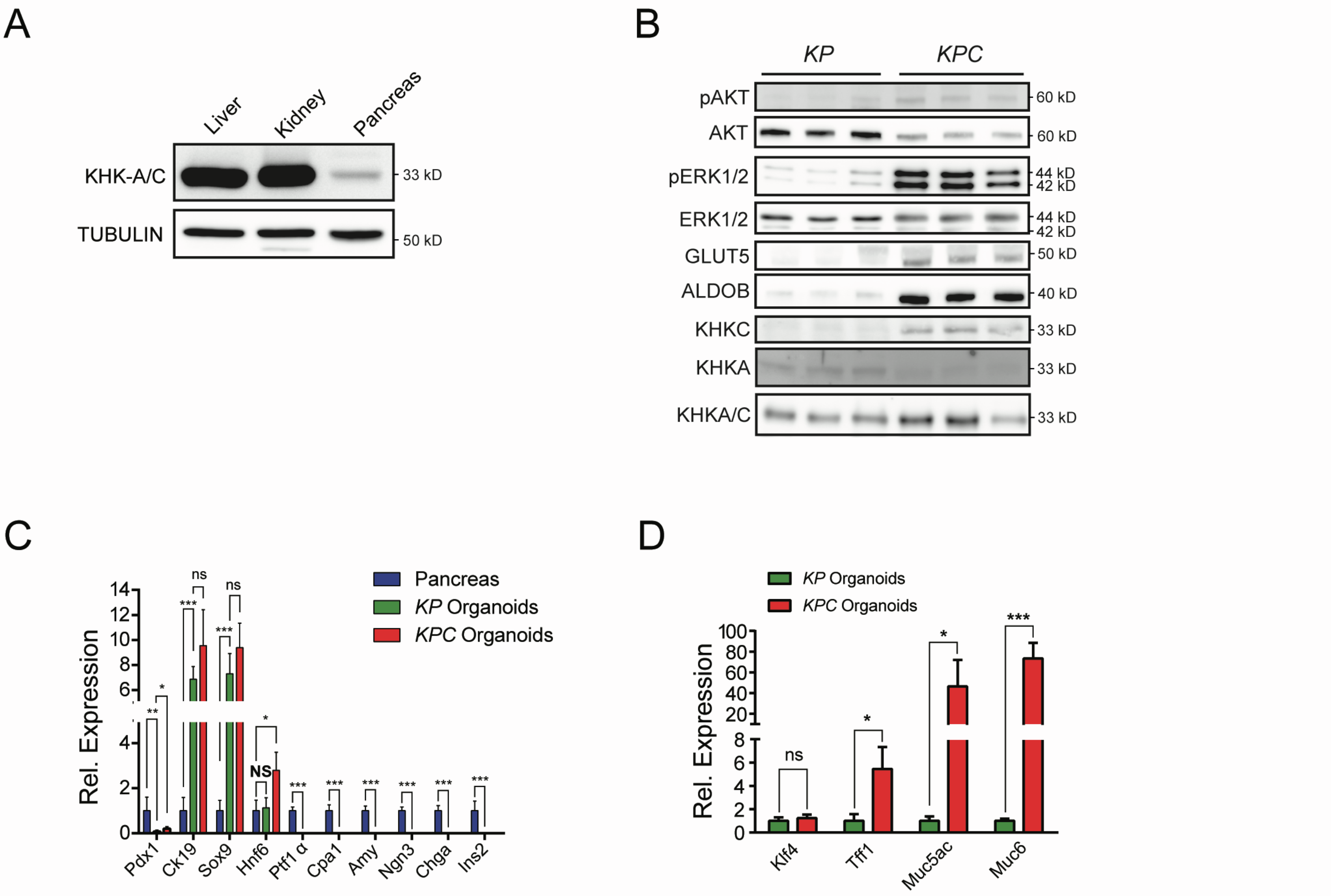

Figure S1. Increased fructolytic and decreased pancreatic lineage expression in KPC organoids. Related to Figure 1.

(**A**) Representative Western blot showing KHK protein levels from normal mouse liver, kidney and pancreas.

(**B**) Representative Western blot analysis of mouse pancreata from KP control versus KPC PDAC tumors.

(**C**) Relative transcript levels of ductal (Pdx1, Ck19, Sox9, and Hnf6), acinar (Ptf1α, Cpa1, and Amy), and endocrine (Ngn3, Chga, and Ins2) lineage markers in pancreas, KP and KPC organoids, n=3.

(**D**) Relative transcript levels of PanIN lesion marker genes (Klf4, Tff1, Muc5ac and Muc6) in KP and KPC organoids.

The p-values were determined by Student's t-test (unpaired two-tailed), n.s. (non-significant), * P<0.05, **p<0.01 and ***p<0.001, ****p<0.0001. Data are represented as Mean ± SEM.

Figure 2

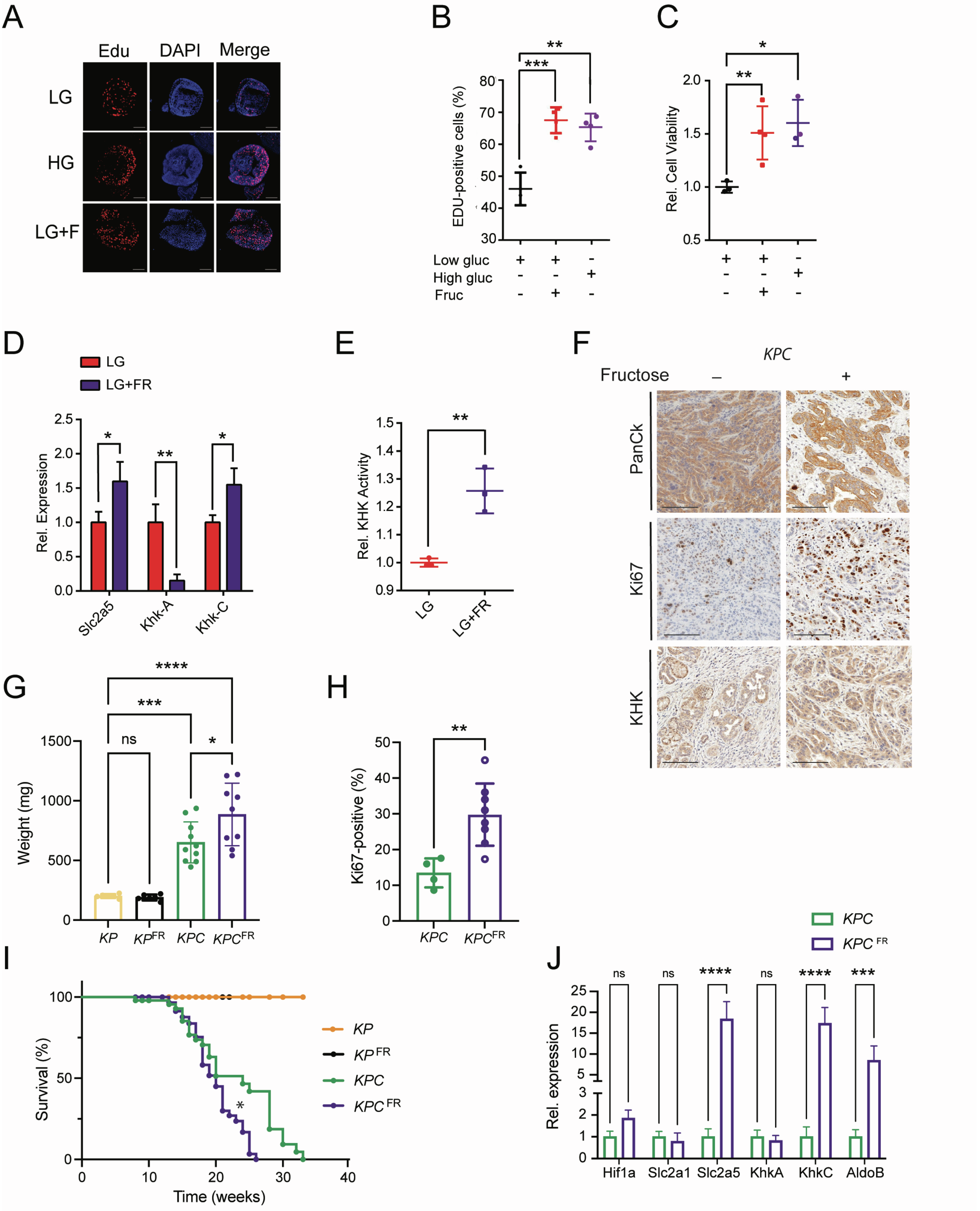
A
Edu
DAPI
Merge
LG
HG
LG+F
B
EDU-positive cells (%)
Low gluc
High gluc
Fruc
C
Rel. Cell Viability
D
LG
LG+FR
Rel. Expression
Slc2a5
Khk-A
Khk-C
E
Rel. KHK Activity
LG
LG+FR
F
KPC
Fructose
PanCk
Ki67
KHK
G
Weight (mg)
KP
KPFR
KPC
KPCFR
H
Ki67-positive (%)
KPC
KPCFR
I
Survival (%)
Time (weeks)
KP
KPFR
KPC
KPCFR
J
KPC
KPC FR
Rel. expression
Hif1a
Slc2a1
Slc2a5
KhkA
KhkC
AldoB

Figure S2. High fructose diet increases tumor growth and reduces survival. Related to Figure 2

(**A, B**) cell viability measurements (left) and cell number counting (right) of mouse *KP* (A) or mouse *KPC* organoids (B) exposed to indicated concentration of fructose, n=3. One-way ANOVA followed by a Tukey's multiple comparison post-test.

(**C**) Assessment of plasma fructose levels in *KP* and *KPC* mice upon 25% of fructose diet, n=34 in total. One-way ANOVA followed by a Tukey's multiple comparison post-test.

(**D**) Gross anatomy of pancreata of *KP* and *KPC* mice with and without 25% of fructose in drinking water.

(**E**) Percentage of survival of *KP* and KPC^{mut} mice with or without 25% of fructose diet (FR) for 10 weeks. Kaplan-Meier survival curves were compared by Mantel-Cox log-rank test; n=152.

(**F**) Representative images of H&E and Ki67 stainings from mouse *KPC* tumors, with and without 25% of fructose in drinking water.

(**G**) Quantification as Ki67-positive cells/field of KPC^{mut} versus KPC^{mut} FR tumors with and without 25% of fructose (FR) in drinking water. n= 4

(**H**) Tumors weight endpoint (g) from KPC^{mut} tumors of mice treated with 25% of fructose diet (FR) for 10 weeks, n= 8–11. One-way ANOVA followed by a Tukey's multiple comparison post-test.

(**I**) Quantification of of PanINs and PDAC progression in mice of indicated genotype and diet, n= 13.

Data are represented as Mean ± SEM. The p-values were determined by ANOVA multiple comparison test (Tukey's test), n.s. (non-significant), * $P<0.05$, ** $p<0.01$ and *** $p<0.001$, **** $p<0.0001$.

Figure S3

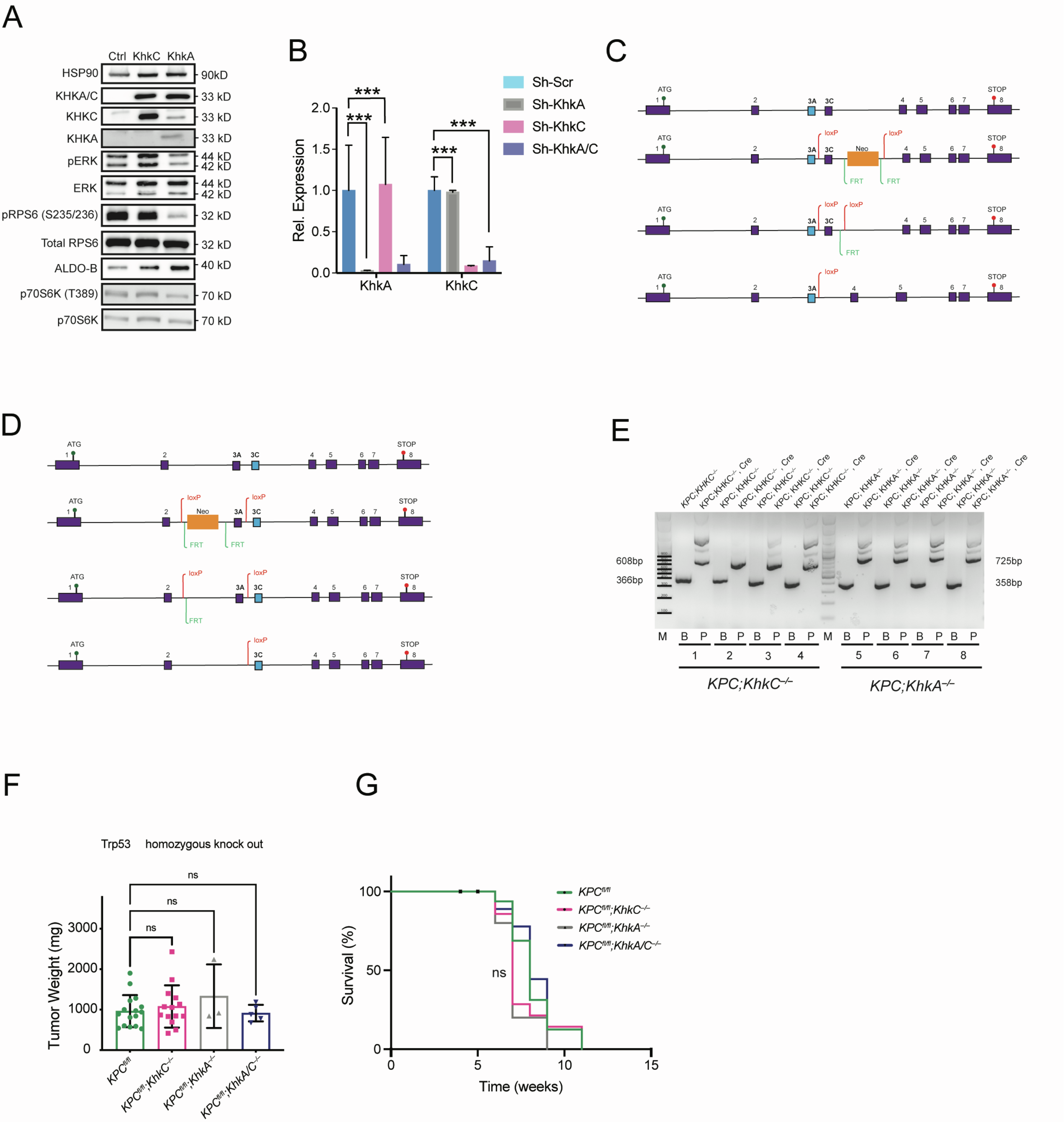
A
Ctrl KhkC KhkA
HSP90
KHKA/C
KHKC
KHKA
pERK
ERK
pRPS6 (S235/236)
Total RPS6
ALDO-B
p70S6K (T389)
p70S6K
90kD
33 kD
44 kD
42 kD
32 kD
40 kD
70 kD
B
Rel. Expression
KhkA
KhkC
Sh-Scr
Sh-KhkA
Sh-KhkC
Sh-KhkA/C
***
C
ATG
STOP
loxP
FRT
Neo
D
E
608bp
366bp
725bp
358bp
M B P
KPC;KhkC-/-
KPC;KhkA-/-
F
Trp53 homozygous knock out
Tumor Weight (mg)
ns
G
Survival (%)
Time (weeks)
ns

Figure S3. Generation of mice with isoform-specific KHK inactivation. Related to Figure 3

(**A**) Representative images of KHK-A or KHK-C overexpression in *KPC* cells followed by immunoblot analysis of MAPK (p-ERK) and mTOR (p70S6K, p70S6K (T389) and pS6 (s235/236) and total pS6) pathways, n=3.

(**B**) Bar graph showing the relative transcript levels of Khk-A and Khk-C upon shScr, shKhkA, shKhkC and shKhkA/C infections in *KPC* cells, n=3.

(**C**) Schematic description of engineered loxP elements allowing the pancreas-specific *KhkC* deletion after Cre expression under the promoter *p48/Ptf1*α.

(**D**) Schematic illustration of engineered loxP elements allowing the pancreas-specific *KhkA* deletion after Cre expression under the *p48/Ptf1*α promoter.

(**E**) PCR-based identification of mutant *KhkC* and *KhkA* alleles from ear biopsies (B) and pancreas tissues (P) upon crossing with KPC mice. Conditional *Khk-C* floxed alleles are shown at 366 bp and *Khk-C* null alleles at 608 bp (left), the conditional *KhkA* floxed allele is 358 bp and *KhkA* null is 725 bp (right). M: molecular weight marker. Numbers indicate individual animals.

(**F**) Tumors weight at 8 weeks of age (mg) from KPC mouse tumors carrying the total knock out for *Trp53* (*Trp53*$^{-/-}$) and knock out for *KhkC*, *KhkA* or *KhkA/C* respectively. ANOVA multiple comparison test (Tukey's test) n=38.

(**G**) Percentage of survival of *KPC*$^{fl/fl}$*, KPC*$^{fl/fl}$*,KhkC*$^{-/-}$*; KPC*$^{fl/fl}$*,KhkA*$^{-/-}$*; KPC*$^{fl/fl}$*,KhkA/C*$^{-/-}$ mice in *Trp53*$^{-/-}$ background, n=68. Kaplan-Meier survival curves were compared by Mantel-Cox log-rank test.

Data are represented as Mean ± SEM.The p-values were determined by ANOVA multiple comparison test (Tukey's test), n.s. (non-significant), *P<0.05, **p<0.01 and ***p<0.001, ****p<0.0001.

Figure S4

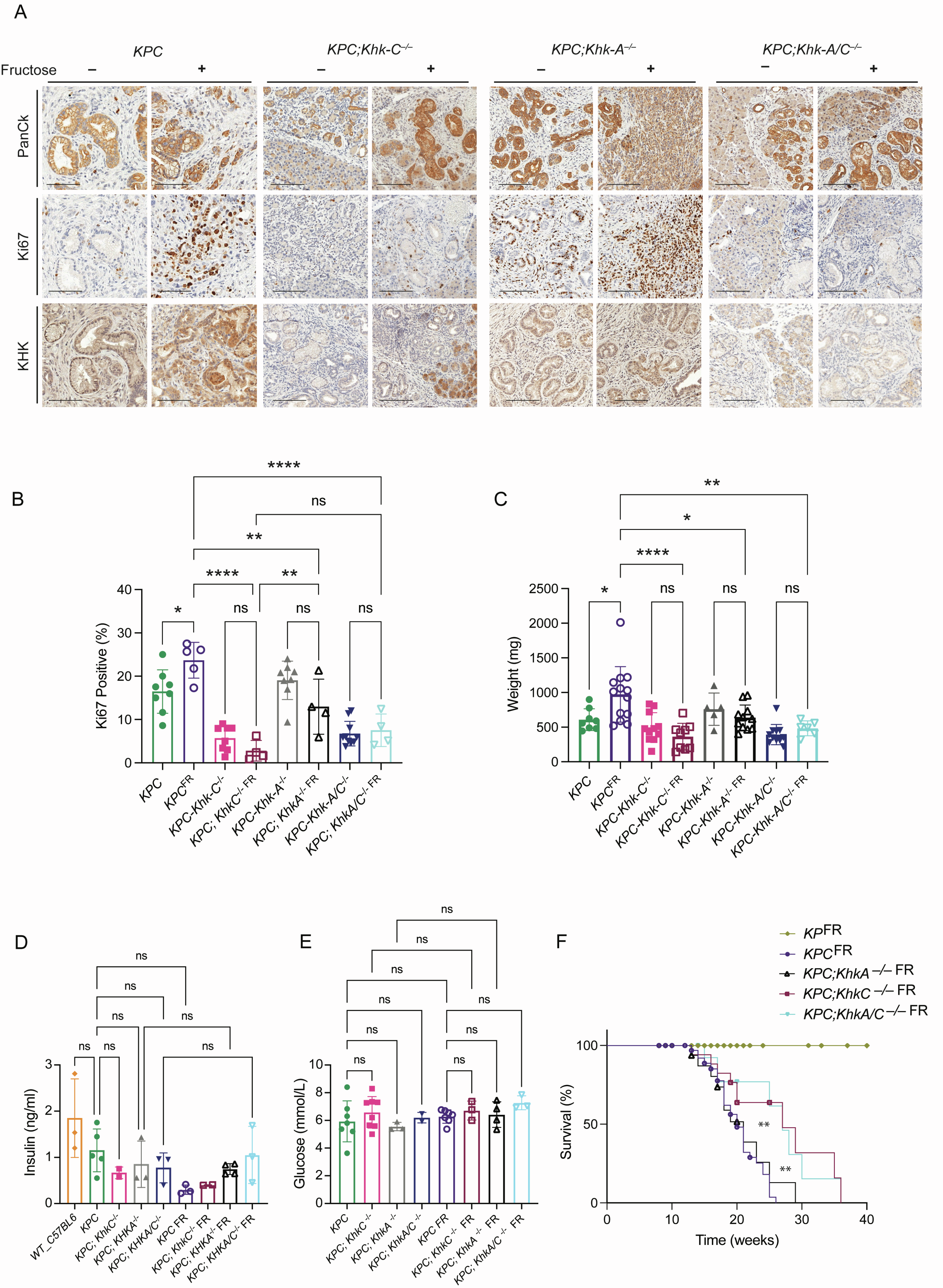
A
KPC
KPC;Khk-C−/−
KPC;Khk-A−/−
KPC;Khk-A/C−/−
Fructose
PanCk
Ki67
KHK
B
Ki67 Positive (%)
KPC
KPC^FR
KPC-Khk-C-/-
KPC; KhkC-/- FR
KPC-Khk-A-/-
KPC; KhkA-/- FR
KPC-Khk-A/C-/-
KPC; KhkA/C-/- FR
C
Weight (mg)
KPC-Khk-C-/- FR
KPC-Khk-A-/- FR
KPC-Khk-A/C-/- FR
D
Insulin (ng/ml)
WT_C57BL6
KPC; KhkC-/-
KPC; KHKA-/-
KPC; KHKA/C-/-
KPC FR
KPC; KhkC-/- FR
KPC; KHKA-/- FR
KPC; KHKA/C-/- FR
E
Glucose (mmol/L)
KPC; KhkA -/-
KPC; KhkA/C -/-
KPC; KhkA -/- FR
F
KP^FR
KPC;KhkA −/− FR
KPC;KhkC −/− FR
KPC;KhkA/C −/− FR
Survival (%)
Time (weeks)

Figure S4. Decreased tumor growth and increased survival in *KPC,KhkC*$^{-/-}$ fed a chow or high fructose diet. Related to Figure 4

(**A**) Representative IHC images of PanCK, Ki67 and KHK stainings in *KPC*, *KPC;KhkC*$^{-/-}$; *KPC;KhkA*$^{-/-}$; *KPC;KhkA/C*$^{-/-}$ mice upon high fructose diet (25% in drinking water) for 10 weeks. Scale bar: 100 μm.

(**B**) Percentage of Ki67 positivity in *KPC*, *KPC;KhkC*$^{-/-}$; *KPC;KhkA*$^{-/-}$; *KPC;KhkA/C*$^{-/-}$ mice treated with 25% of fructose (FR) for 10 weeks, n=51. One-way ANOVA followed by a Tukey's multiple comparison post-test.

(**C**) Tumors weight endpoint (mg) from *KPC*, *KPC;KhkC*$^{-/-}$; *KPC;KhkA*$^{-/-}$; *KPC;KhkA/C*$^{-/-}$ mice treated with 25% fructose (FR) for 10 weeks, n=68. One-way ANOVA followed by a Tukey's multiple comparison post-test.

(**D**) Endpoint insulin concentrations (ng/ml) analyzed in *KPC*, *KPC;KhkC*$^{-/-}$; *KPC;KhkA*$^{-/-}$; *KPC;KhkA/C*$^{-/-}$ mice with and without 25% of fructose (FR), n=28. One-way ANOVA followed by a Tukey's multiple comparison post-test.

(**E**) Endpoint glucose concentration (mmol/l) analyzed in *KPC*, *KPC;KhkC*$^{-/-}$; *KPC;KhkA*$^{-/-}$; *KPC;KhkA/C*$^{-/-}$ mice with and without 25% of fructose (FR), n=37. One-way ANOVA followed by a Tukey's multiple comparison post-test.

(**F**) Percentage of survival of *KPC*, *KPC;KhkC*$^{-/-}$; *KPC;KhkA*$^{-/-}$; *KPC;KhkA/C*$^{-/-}$ mice upon 25% of fructose (FR). Kaplan-Meier survival curves were compared by Mantel-Cox log-rank test; n=149 in total

Data are represented as Mean ± SEM. The p values were determined by One-way ANOVA multiple comparison test (Tukey's test), n.s. (non-significant), *P<0.05, **p<0.01 and ***p<0.001, ****p<0.0001.

Figure S5

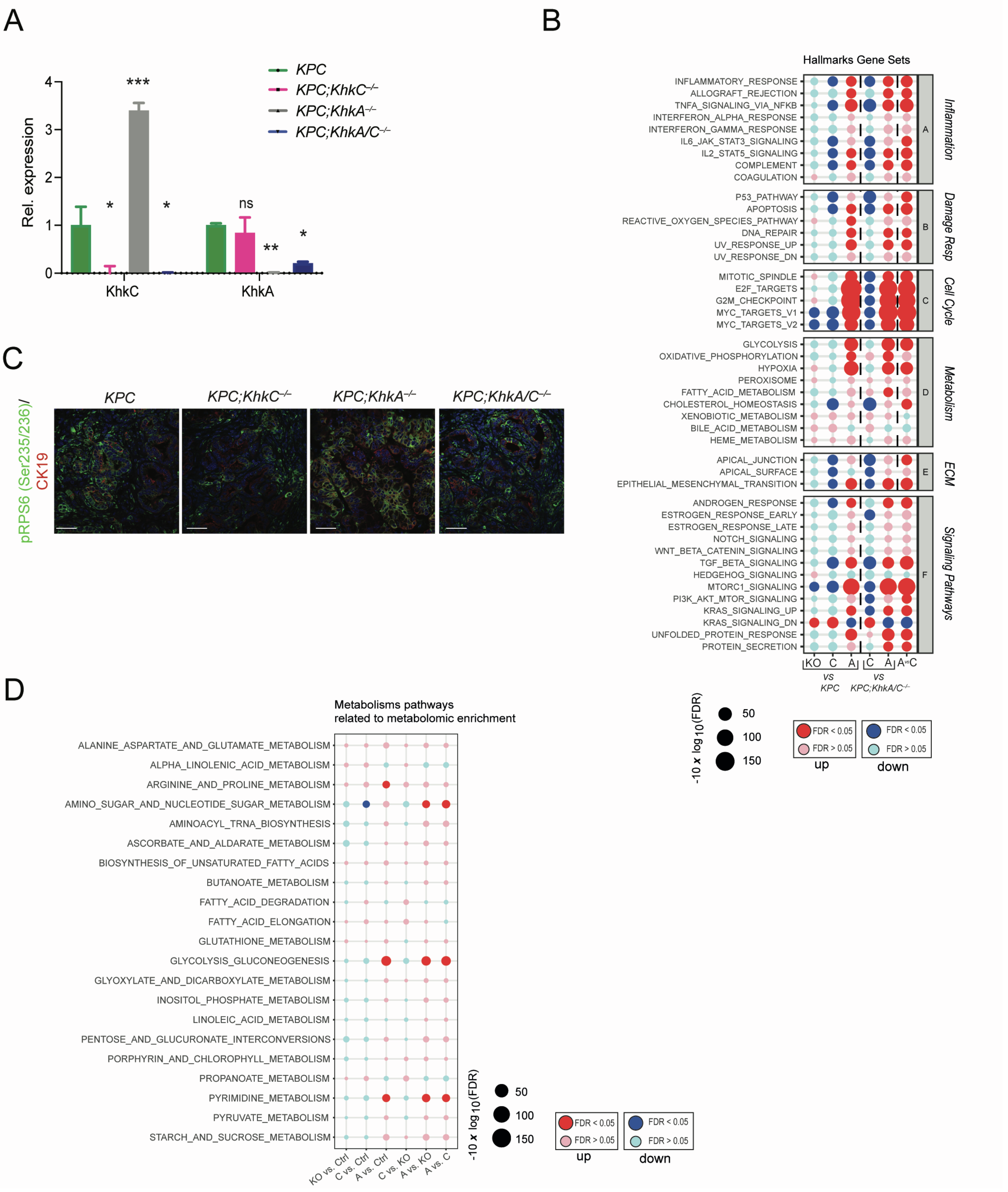

A
B
C
D
Rel. expression
KPC
KPC;KhkC−/−
KPC;KhkA−/−
KPC;KhkA/C−/−
KhkC
KhkA
ns
Hallmarks Gene Sets
Inflammation
Damage Resp
Cell Cycle
Metabolism
ECM
Signaling Pathways
pRPS6 (Ser235/236)/
CK19
Metabolisms pathways
related to metabolomic enrichment
-10 x log10(FDR)
FDR < 0.05
FDR > 0.05
up
down

Figure S5. Metabolic pathway analysis of tumor cells with global and isoform-specific *Khk* deletion. Related to Figure 5

(**A**) Relative transcript levels of KhkC and KhkA isoforms from *KPC*, *KPC;KhkC*$^{-/-}$; *KPC;KhkA*$^{-/-}$; *KPC;KhkA/C*$^{-/-}$ Epcam+ mouse tumors-derived cells.

(**B**) R Gene-set enrichment (GSEA hallmark pathway) analysis from Epcam+ CD45^{-} *ex vivo* tumor cells RNA seq. Dot plots represent the changes in GSEA hallmark pathways of *KPC;KhkA/C*$^{-/-}$ (KO), *KPC;KhkC*$^{-/-}$ (C) and *KPC;KhkA*$^{-/-}$ (A) versus *KPC, KPC;KhkC*$^{-/-}$ (C) and *KPC;KhkA*$^{-/-}$ (A) versus *KPC;KhkA/C*$^{-/-}$ and *KPC;KhkA*$^{-/-}$ (A) versus *KPC;KhkC*$^{-/-}$ (C). (*KPC*: n=3; *KhkA/C*$^{-/-}$: n=3; *KhkC*$^{-/-}$: n=2; *KhkA*$^{-/-}$: n=2). P values are expressed as $-10 \times \log_{10}$ FDR (FDR-adjusted). Upregulated: red; downregulated: blue.

(**C**) Images of immunofluorescence stainings (IF) showing the expression of pRS6 (Ser235/236), CK19 and DAPI in all four tumor sections. Scale bar: 45 μm.

(**D**) Dot plots represent KEGG analysis of metabolism pathways from transcriptomics analysis of *ex vivo* Epcam+; CD45- tumor cells. (KPC: n=3; *KhkA/C*$^{-/-}$: n=3; *KhkC*$^{-/-}$: n=2; *KhkA*$^{-/-}$: n=2). P values are expressed as $-10 \times \log$ 10FDR (FDR-adjusted). Upregulated: red; downregulated: blue.

Data S1/Methods S1: Oligonucleotide sequences used for qPCR, shRNA, and genotyping of mutant mice. Related to Figures 1–4.

Mouse qPCR primers		
**Species**	**Primers**	**Sequences (5' - 3')**
Mouse	*Hif1α*_Fwd	TCTCGGCGAAGCAAAGAGTC
Mouse	*Hif1α*_Rev	AGCCATCTAGGGCTTTCAGATAA
Mouse	*KhkA/C*_Fwd	AGGTCGATCTGACCCGGTT
Mouse	*KhkA/C*_Rev	TCA CG G G GC TTC TC T A TC TCC
Mouse	*Khk-A*_Fwd	AACTCCTGCACTGTCCTTTC
Mouse	*Khk-A*_Rev	GACCACATATCGTAAGTCCACAG
Mouse	*Khk-C*_Fwd	GGCTCCCGTACCATTATACTC
Mouse	*Khk-C*_Rev	CAGATCGACCTTCTCAAAGTCC
Mouse	*Slc2a1*_Fwd	CAGTTCGGCTATAACACTGGTG
Mouse	*Slc2a1*_Rev	GCCCCCGACAGAGAAGATG
Mouse	*Hk2*_Fwd	ATGATCGCCTGCTTATTCACG
Mouse	*Hk2*_Rev	CGCCTAGAAATCTCCAGAAGGG
Mouse	*Aldob*_Fwd	GAAACCGCCTGCAAAGGATAA
Mouse	*Aldob*_Rev	GAGGGTCTCGTGGAAAAGGAT
Mouse	*Slc2a5*_Fwd	CCAATATGGGTACAACGTAGCTG
Mouse	*Slc2a5*_Rev	CCAATATGGGTACAACGTAGCTG
Mouse	*β-Actin*_Fwd	GTGACGTTGACATCCGTAAAGA
Mouse	*β-Actin*_Rev	GCCGGACTCATCGTACTCC
Mouse	*Pdx1*_Fwd	CCCGGACCTTTCCCGAATGG
Mouse	*Pdx1*_Rev	CTCGGGTTCCGCTGTGTAAGC
Mouse	*Ck19*_Fwd	GTTCAGTACGCATTGGGTCAG
Mouse	*Ck19*_Rev	GAGGACGAGGTCACGAAGC
Mouse	*Sox9*_Fwd	CTCCTAATGCTATCTTCAAG
Mouse	*Sox9*_Rev	GCTTCAGATCAACTTTGC
Mouse	*Hnf6*_Fwd	GGCTATGCCCACCGACAAG
Mouse	*Hnf6*_Rev	GCCCTGAATTACTTCCATTGCT
Mouse	*Ptf1a*_Fwd	TCCCATCCCCTTACTTTGATGA
Mouse	*Ptf1a*_Rev	CGGTAGCAGTATTCGTGTAGC
Mouse	*Cpa1*_Fwd	GTCTTCGGCAATGAGAACTTTGT
Mouse	*Cpa1*_Rev	GGAAGGGCACTCGAACATCG
Mouse	*Amy*_Fwd	GCAAAATGAAGTTCGTTCTGC
Mouse	*Amy*_Rev	TGATGCTTAATCCAAATCTC
Mouse	*Ngn3*_Fwd	CCAAGAGCGAGTTGGCACT
Mouse	*Ngn3*_Rev	CGGGCCATAGAAGCTGTGG
Mouse	*Chga*_Fwd	CCAAGGTGATGAAGTGCGTC
Mouse	*Chga*_Rev	GGTGTCGCAGGATAGAGAGGA
Mouse	*Ins2*_Fwd	GCTTCTTCTACACACCCATGTC
Mouse	*Ins2*_Rev	AGCACTGATCTACAATGCCAC
Mouse	*Klf4*_Fwd	GGCGAGTCTGACATGGCTG
Mouse	*Klf4*_Rev	GGCGAGTCTGACATGGCTG
Mouse	*Muc5ac*_Fwd	CAGGACTCTCTGAAATCGTACCA
Mouse	*Muc5ac*_Rev	GAAGGCTCGTACCACAGGG

Data S1/Methods S1 continued: Oligonucleotide sequences used for qPCR, shRNA, and genotyping of mutant mice. Related to Figures 1–4, continued

Mouse	*Muc6*_Fwd	CACCTTTGACGGCCATGAGTA
Mouse	*Muc6*_Rev	GGTGTAGGGCAGGCTAACAA
Mouse	*Tff1*_Fwd	AGCACAAGGTGATCTGTGTCC
Mouse	*Tff1*_Rev	GAAGCCACAATTTATCCTCTCCC
Mouse	*Slc2a2*_Fwd	TCAGAAGACAAGATCACCGGA
Mouse	*Slc2a2*_Rev	GCTGGTGTGACTGTAAGTGGG
Mouse	*Hk1*_Fwd	CGG AAT GGG GAG CCT TTG G
Mouse	*Hk1*_Rev	GCC TTC CTT ATC CGT TTC AAT GG
Mouse	*Myc*_Fwd	ATGCCCCTCAACGTGAACTTC
Mouse	*Myc*_Rev	CGCAACATAGGATGGAGAGCA
Mouse	*Got1*_Fwd	GCG CCT CCA TCA GTC TTT G
Mouse	*Got1*_Rev	ATT CAT CTG TGC GGT ACG CTC
Mouse	*Mdh2*_Fwd	TTG GGC AAC CCC TTT CAC TC
Mouse	*Mdh2*_Rev	GCC TTT CAC ATT TGC TCT GGT C
Mouse	*Pkm*_Fwd	GCC GCC TGG ACA TTG ACT C
Mouse	*Pkm*_ Rev	CCA TGA GAG AAA TTC AGC CGA G
Mouse	*Acly*_Fwd	ACC CTT TCA CTG GGG ATC ACA
Mouse	*Acly*_Rev	GAC AGG GAT CAG GAT TTC CTT G
Mouse	*Cpt1a*_Fwd	CTC CGC CTG AGC CAT GAA G
Mouse	*Cpt1a*_Rev	CAC CAG TGA TGA TGC CAT TCT
Mouse	*Fasn*_Fwd	GGA GGT GGT GAT AGC CGG TAT
Mouse	*Fasn*_Rev	TGG GTA ATC CAT AGA GCC CAG
Mouse	*Acaca*_Fwd	GAT GAA CCA TCT CCG TTG GC
Mouse	*Acaca*_Rev	GAC CCA ATT ATG AAT CGG GAG TG
Mouse	*Srebf1*_Fwd	TGA CCC GGC TAT TCC GTG A
Mouse	*Srebf1*_Rev	CTG GGC TGA GCA ATA CAG TTC
Mouse	*Srebf2*_Fwd	GCA GCA ACG GGA CCA TTC T
Mouse	*Srebf2*_Rev	CCC CAT GAC TAA GTC CTT CAA CT
Mouse	*Sqle*_Fwd	ATA AGA AAT GCG GGG ATG TCA C
Mouse	*Sqle*_Rev	ATA TCC GAG AAG GCA GCG AAC
Mouse	*Acacb*_Fwd	CCT TTG GCA ACA AGC AAG GTA
Mouse	*Acacb*_Rev	AGT CGT ACA CAT AGG TGG TCC
Mouse	*Accs1*_Fwd	GTT TGG GAC ACT CCT TAC CAT AC
Mouse	*Accs1*_Rev	AGG CAG TTG ACA GAC ACA TTC
Mouse	*Accs2*_Fwd	AAA CAC GCT CAG TAG CAC CAC
Mouse	*Accs2*_Rev	AGC CAA GTA GGA AGC TCT CTC
Mouse	*Cycloph.*_Fwd	GAG CTG TTT GCA GAC AAA GTT C
Mouse	*Cycloph.*_Rev	CCC TGG CAC ATG AAT CCT GG
Mouse	*18S*_Fwd	GTT CCG ACC ATA AAC GAT GCC
Mouse	*18S*_Rev	TGG TGG TGC CCT TCC GTC AAT
Mouse	sh-RNA KhkA-1 Fwd	5'CCGGGGACTTACGATATGTGGTCCT CTCGAGAGGACCACATATCGTAAGTC C TTTTTG-3'
Mouse	sh-RNA KhkA-1 Rev	5'AATTCAAAAAGGACTTACGATATGT GGTCCTCTCGAGAGGACCACATATCG TAAGTCC-3'

Data S1/Methods S1 continued: Oligonucleotide sequences used for qPCR, shRNA, and genotyping of mutant mice. Related to Figures 1–4, continued

Mouse	sh-RNA KhkA-2 Fwd	5'CCGGGCCAACATTCTGTGGACTTCTCGAGAAGTCCACAGAATGTTGGCTTTTTG-3'
Mouse	Sh-RNA KhkA-2 Rev	5'CCGGGCTCCCGTACCATTATACTCTCTCGAGAGAGTATAATGGTACGGGAGC-3'
Mouse	Sh-RNA KhkC-1 Fwd	5'CCGGGGCTCCCGTACCATTATACTCCTCGAGGAGTATAATGGTACGGGAGCCTTTTTG-3'
Mouse	Sh-RNA KhkC-1 Rev	5'AATTCAAAAAGGCTCCCGTACCATTATACTCCTCGAGGAGTATAATGGTACGGGAGCC-3'
Mouse	Sh-RNA KhkC-2 Fwd	5'CCGGGCTCCCGTACCATTATACTCTCTCGAGAGAGTATAATGGTACGGGAGC TTTTTG-3'
Mouse	Sh-RNA KhkC-2 Rev	5'AATTCAAAAAGCTCCCGTACCATTATACTCTCTCGAGAGAGTATAATGGTACGGGAGC -3'
**Human qRT-PCR primer sequences**		
**Species**	**Primers**	**Sequences (5' – 3')**
Human	*KHK-A*_Fwd	GCTATTCTGTGGACCTACGCT
Human	*KHK-A*_Rev	CAATGTGGATCCACTTGAACTG
Human	*KHK-C*_Fwd	CTCCTGCTGCATCATCAAC
Human	*KHK-C*_Rev	CAATGTGGATCCACTTGAACTG
Human	*SLC2A5*_Fwd	GAGGCTGACGCTTGTGCTT
Human	*SLC2A5*_Rev	CCACGTTGTACCCATACTGGA
Human	*ALDOB*_Fwd	GGCAGTTCCGAGAAATCCTCT
Human	*ALDOB*_Rev	CTCCTTGGTCTAACTTGATTCCC
Human	*β-ACTIN*_Fwd	CATGTACGTTGCTATCCAGGC
Human	*β-ACTIN*_Rev	CTCCTTAATGTCACGCACGAT
**Mouse genotyping primer sequences**		
**Species**	**Primers**	**Sequences (5' – 3')**
Mouse	*Kras G12D*_Fwd	CTA GCC ACC ATG GCT TGA GT
Mouse	*Kras G12D*_Rev	TCC GAA TTC AGT GAC TAC AGA TG
Mouse	*Lox P53*_Fwd	GGT TAA ACC CAG CTT GAC CA
Mouse	*Lox P53*_Rev	GGA GG C A GA GAC AG T T GG AG
Mouse	*P53 mut*_Fwd	AGC TAG CCA CCA TGG CTT GAG TAA GTC TGC A
Mouse	*P53 mut*_Rev	CTT GGA GAC ATA GCC ACA CTG
Mouse	*p48*_Fwd (Common Cre)	GAGCAGCCCATTCGTCCT
Mouse	*p48*_Rev (transgene Cre)	GGTTCTTGCGAACCTCATCA
Mouse	*Khk_WT*_Fwd	AGG TCG ATC TGA CCC GGT T
Mouse	*Khk_WT*_Rev	TCA CGG GGC TTC TCT ATC TCC
Mouse	*KhkΔ*_Fwd	TTCTTCTCTGAGGCCCATGT
Mouse	*KhkΔ*_Rev	TCACACAATGCCATCAAACC
Mouse	*KhkA floxed*_Fwd	GCCTTAACTCTACCTCTGTCTAGC

Data S1/Methods S1 continued: Oligonucleotide sequences used for qPCR, shRNA, and genotyping of mutant mice. Related to Figures 1–4, continued

Mouse	*KhkA floxed*_Rev	GAAGAGTTTGGGGGACTACCAG
Mouse	*KhkC floxed*_Fwd	CAAGGTGCTGATTGGCTAATTATG
Mouse	*KhkC floxed*_Rev	CTACCACAACCCACCAATCAGTC
